# Quantitative live imaging of Venus::BMAL1 in a mouse model reveals complex dynamics of the master circadian clock regulator

**DOI:** 10.1371/journal.pgen.1008729

**Published:** 2020-04-30

**Authors:** Nan Yang, Nicola J. Smyllie, Honor Morris, Cátia F. Gonçalves, Michal Dudek, Dharshika R. J. Pathiranage, Johanna E. Chesham, Antony Adamson, David G. Spiller, Egor Zindy, James Bagnall, Neil Humphreys, Judith Hoyland, Andrew S. I. Loudon, Michael H. Hastings, Qing-Jun Meng

**Affiliations:** 1 Wellcome Centre for Cell Matrix Research, University of Manchester, Manchester, United Kingdom; 2 Faculty of Biology, Medicine and Health, University of Manchester, Manchester, United Kingdom; 3 Division of Neurobiology, Medical Research Council Laboratory of Molecular Biology, Francis Crick Avenue, Cambridge, United Kingdom; 4 NIHR Manchester Musculoskeletal Biomedical Research Centre, Manchester University NHS Foundation Trust, Manchester Academic Health Science Centre, Manchester, United Kingdom; Charité - Universitätsmedizin Berlin, GERMANY

## Abstract

Evolutionarily conserved circadian clocks generate 24-hour rhythms in physiology and behaviour that adapt organisms to their daily and seasonal environments. In mammals, the suprachiasmatic nucleus (SCN) of the hypothalamus is the principal co-ordinator of the cell-autonomous clocks distributed across all major tissues. The importance of robust daily rhythms is highlighted by experimental and epidemiological associations between circadian disruption and human diseases. BMAL1 (a bHLH-PAS domain-containing transcription factor) is the master positive regulator within the transcriptional-translational feedback loops (TTFLs) that cell-autonomously define circadian time. It drives transcription of the negative regulators *Period* and *Cryptochrome* alongside numerous clock output genes, and thereby powers circadian time-keeping. Because deletion of *Bmal1* alone is sufficient to eliminate circadian rhythms in cells and the whole animal it has been widely used as a model for molecular disruption of circadian rhythms, revealing essential, tissue-specific roles of BMAL1 in, for example, the brain, liver and the musculoskeletal system. Moreover, BMAL1 has clock-independent functions that influence ageing and protein translation. Despite the essential role of BMAL1 in circadian time-keeping, direct measures of its intra-cellular behaviour are still lacking. To fill this knowledge-gap, we used CRISPR Cas9 to generate a mouse expressing a knock-in fluorescent fusion of endogenous BMAL1 protein (Venus::BMAL1) for quantitative live imaging in physiological settings. The *Bmal1^Venus^* mouse model enabled us to visualise and quantify the daily behaviour of this core clock factor in central (SCN) and peripheral clocks, with single-cell resolution that revealed its circadian expression, anti-phasic to negative regulators, nuclear-cytoplasmic mobility and molecular abundance.

## Author summary

Cell-autonomous circadian clocks are transcriptional/translational feedback loops that co-ordinate almost all mammalian physiology and behaviour. Although their genetic basis is well understood, we are largely ignorant of the natural behaviour of clock proteins and how they work within these loops. This is particularly true for the essential transcriptional activator BMAL1. To address this, we created and validated a mouse carrying a fully functional knock-in allele that encodes a fluorescent fusion of BMAL1 (Venus::BMAL1). Quantitative live imaging in tissue explants and cells, including the central clock of the suprachiasmatic nucleus (SCN), revealed the circadian expression, nuclear-cytoplasmic mobility, fast kinetics and surprisingly low molecular abundance of endogenous BMAL1, providing significant quantitative insights into the intracellular mechanisms of circadian timing at single-cell resolution.

## Introduction

Circadian clocks are evolutionarily conserved time-keeping mechanisms that generate ca. 24-hour rhythms in physiology and behaviour, allowing organisms to anticipate and adapt to their daily and seasonal environments. In mammals, the suprachiasmatic nucleus (SCN) of the hypothalamus is the principal co-ordinator of the innumerable cell-autonomous clocks distributed across all major tissues [1–3]. The genetic decoding of circadian clockworks has been a standout success in revealing the transcriptional/translational feedback loops (TTFL) within cells that define circadian time and temporally co-ordinate the expression and activity of downstream genes and pathways to the 24-hour day [1–4]. Although the genetic basis of the TTFL is well understood, one should not lose sight, however, that the genes are the entry point. We shall only understand circadian mechanisms once we have deciphered how the encoded proteins work in defining ca. 24 hour time: we need to put protein flesh onto the genetic scaffold of the self-sustaining TTFL.

This is particularly true for the essential transcriptional activator BMAL1 (a bHLH-PAS domain-containing transcription factor). In mammals, BMAL1 is the master circadian clock regulator at the core of the TTFL. It drives transcription of the negative regulators Period and Cryptochrome alongside numerous clock output genes, and thereby powers circadian time-keeping [5]. A single gene knockout of BMAL1 in animals is sufficient to eliminate circadian rhythms in cells, tissues and at the whole organism level. As such, BMAL1 knockout has been widely used as a model for molecular disruption of circadian rhythms, revealing essential, tissue-specific roles of BMAL1 in, for example, the brain, liver and the musculoskeletal system [6, 7]. Moreover, BMAL1 has clock-independent functions that influence ageing and protein translation [8, 9]. Despite this key importance, direct measures of the intra-cellular behaviour of BMAL1 in central and peripheral clocks are still lacking.

To address this, we report the design, creation and validation in a mouse of a fully functional knock-in allele that encodes a fluorescent fusion of BMAL1 (Venus::BMAL1). Real-time light microscopy enables quantitative, live imaging over multiple circadian cycles of the behaviour of endogenous BMAL1 in tissue explants and cells, including the central circadian pacemaker of the SCN. Thus, our findings provide new quantitative insights into the molecular intricacy of an essential component of the mammalian circadian clock at single cell level.

## Results and discussion

### Generation and characterization of a fully functional Venus::BMAL1 knock-in mouse.

**Fig 1:**
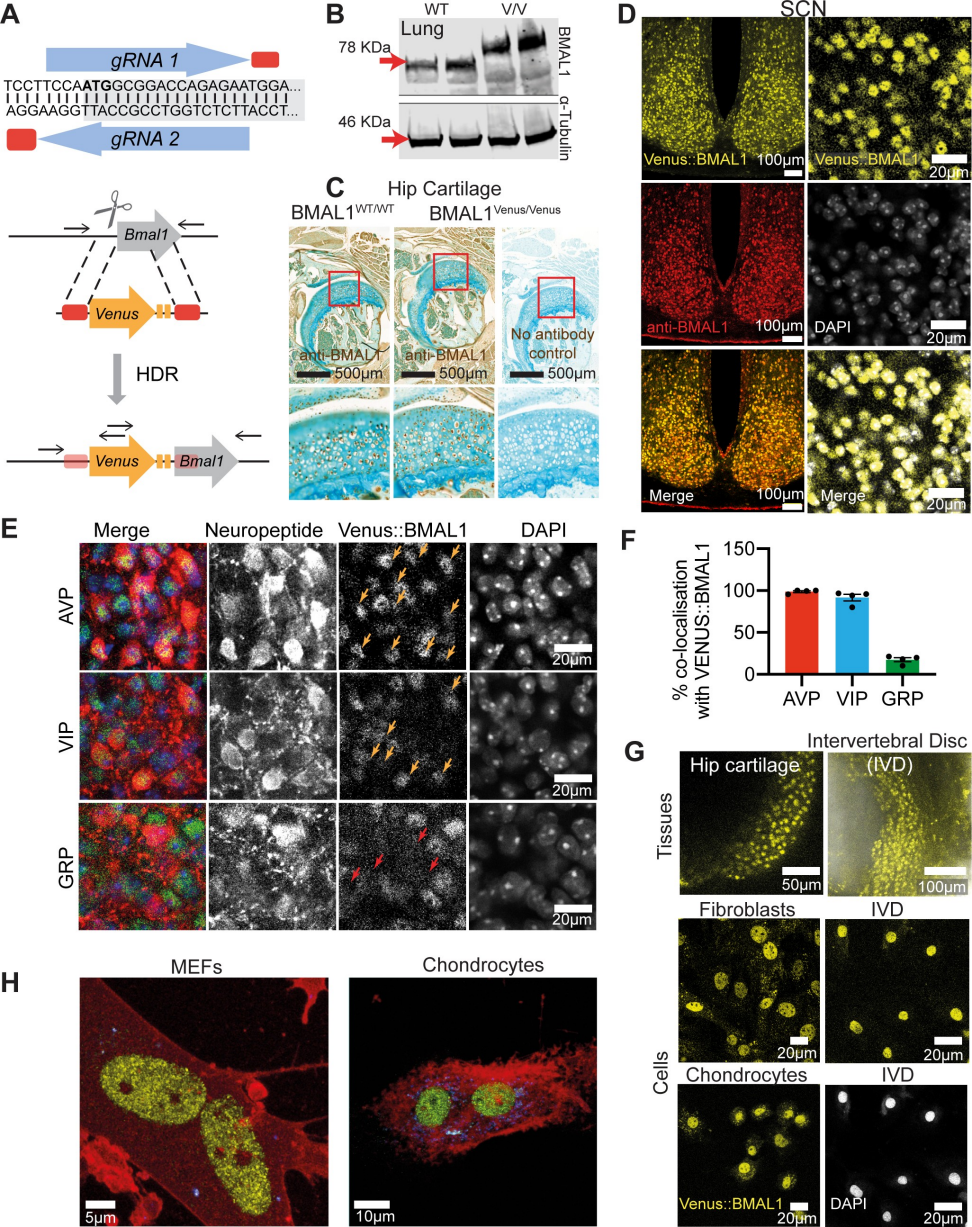
Generation and validation of Venus::BMAL1 mouse. A) sgRNA targeting design across *Bmal1* start codon (ATG in bold, shading indicates gene coding sequence). HDR targeting cassette design, cut site indicated by scissors, corresponding homology arms by red boxes and Venus-linker-linker in yellow. Black arrows show post-HDR allele primers for genotyping. B) WB in mouse lung with anti-BMAL1 antiserum, showing increased molecular weight of Venus::BMAL1 (V/V). C) There was no difference in the pattern of BMAL1 expression (shown by IHC) between Venus and non-Venus mouse hip. “No antibody control” confirms the specificity of the antiserum. D) Top: Representative confocal images of SCN from brain sections taken from Venus::BMAL1 animals. Venus::BMAL1 showed nearly 100% registration with endogenous BMAL1 in the SCN from heterozygous animals. Expression of Venus::BMAL1 (yellow) was revealed by confocal fluorescence imaging and IF with an anti-BMAL1 antibody (red). Bottom: Venus::BMAL1-positive localisation was compared with nuclear staining of DAPI. Venus::BMAL1 was predominantly expressed in the nuclei of cells in the SCN. Scale bars as written in Figure. E) Representative confocal micrographs showing close-up images of cells in fixed SCN sections where Venus::BMAL1 (green) is colocalised with SCN neuropeptides (VIP, AVP and GRP shown in red) by IHC and co-stained with DAPI (blue). Colocalised cells are indicated by arrows shown in orange. Red arrows show gaps in Venus::BMAL1 signal which align with GRP cells. F) Percentage neuropeptide-immunostained cells that co-localise with Venus::BMAL1-positive cells (n = 4 animals). G) Representative Venus::BMAL1 signals in femoral head articular cartilage and intervertebral disc tissues (top) and in primary chondrocyte, fibroblast and IVD cultures (bottom). The IVD preparation was co-stained with DAPI, shown below the fluorescence image. Scale bars as written in Figure. H) Representative visualisation of Venus::BMAL1 fluorescence in MEF and chondrocyte nuclei generated in Imaris from Z-stack spectral imaging. Spectral linear unmixing revealed nuclear localization of Venus::BMAL1, with most of the cytoplasmic fluorescence attributed to auto-fluorescence. Yellow: Venus::BMAL1; Red: CellMask Deep Red Plasma membrane stain; blue: auto-fluorescence.

Because the C-terminus of BMAL1 interacts with Cryptochrome (CRY) and CREB-binding protein to regulate circadian pacemaking [10, 11], we used CRISPR/Cas9 to introduce an N-terminal, in-frame Venus fluorescent tag (Fig 1A). PCR genotyping and sequencing of genomic DNA from the founder line confirmed successful targeting and homology-directed repair (S1A–S1C Fig), and Western Blot (WB) of lung tissue demonstrated the higher molecular weight of BMAL1 from *Bmal1^Venus/Venus^* mice (Fig 1B). The cellular distribution of BMAL1-immunoreactivity (-ir) was comparable between *Bmal1^Venus/Venus^* and *Bmal1^WT/WT^* hip cartilage (Fig 1C and S1D Fig), whilst in the SCN Venus fluorescence was in direct spatial register with BMAL1-ir. Importantly, Venus::BMAL1 co-localised with DAPI (Fig 1D) (Mander’s co-localisation co-efficient = 0.90 ±0.01; n = 4), and thus was strongly nuclear, with cytoplasmic fluorescence much weaker (nuclear:cytoplasmic ratio of 2.8 ±0.2, mean ±SEM; n = 4). Importantly, the expression of canonical SCN neuropeptides; vasoactive intestinal polypeptide (VIP), arginine vasopressin (AVP) and gastrin-releasing peptide (GRP), did not differ between WT and *Bmal1*^*Venus*/*Venus*^ mice (S2A and S2B Fig). Venus::BMAL1 was expressed in the nucleus of nearly all VIP- and AVP-ir cells, whereas fewer than 20% of GRP-ir neurons contained Venus::BMAL1 (Fig 1E and 1F and S2B Fig). A similar absence from GRP cells was noted for PER2::Venus [12], suggesting that GRP cells may lack a cell-autonomous TTFL [13]. BMAL1 has essential homeostatic roles in femoral head articular cartilage (hip) and intervertebral disc (IVD), which demonstrate robust circadian rhythms [14, 15]. In explants and dispersed cultures of both tissues, Venus::BMAL1 fluorescence was expressed in virtually all cells (Fig 1G), and again co-localised with a nuclear marker (DRAQ5) (S2C Fig). Only peri-threshold levels of fluorescence were observed in the cytoplasm. Spectral (lambda) imaging and linear unmixing revealed an even more striking nuclear localisation of Venus::BMAL1, with most of the cytoplasmic fluorescence attributed to auto-fluorescence in both mouse embryonic fibroblasts (MEFs) and chondrocytes (Fig 1H; S1 and S2 Movies). This is consistent with proteomics studies showing that BMAL1 is primarily found in large complexes of clock proteins (including CLOCK) within the nucleus [16]. We conclude that the *Bmal1^Venus^* allele encodes a faithful reporter of endogenous BMAL1 expression and its intra-cellular localisation.

**Fig 2:**
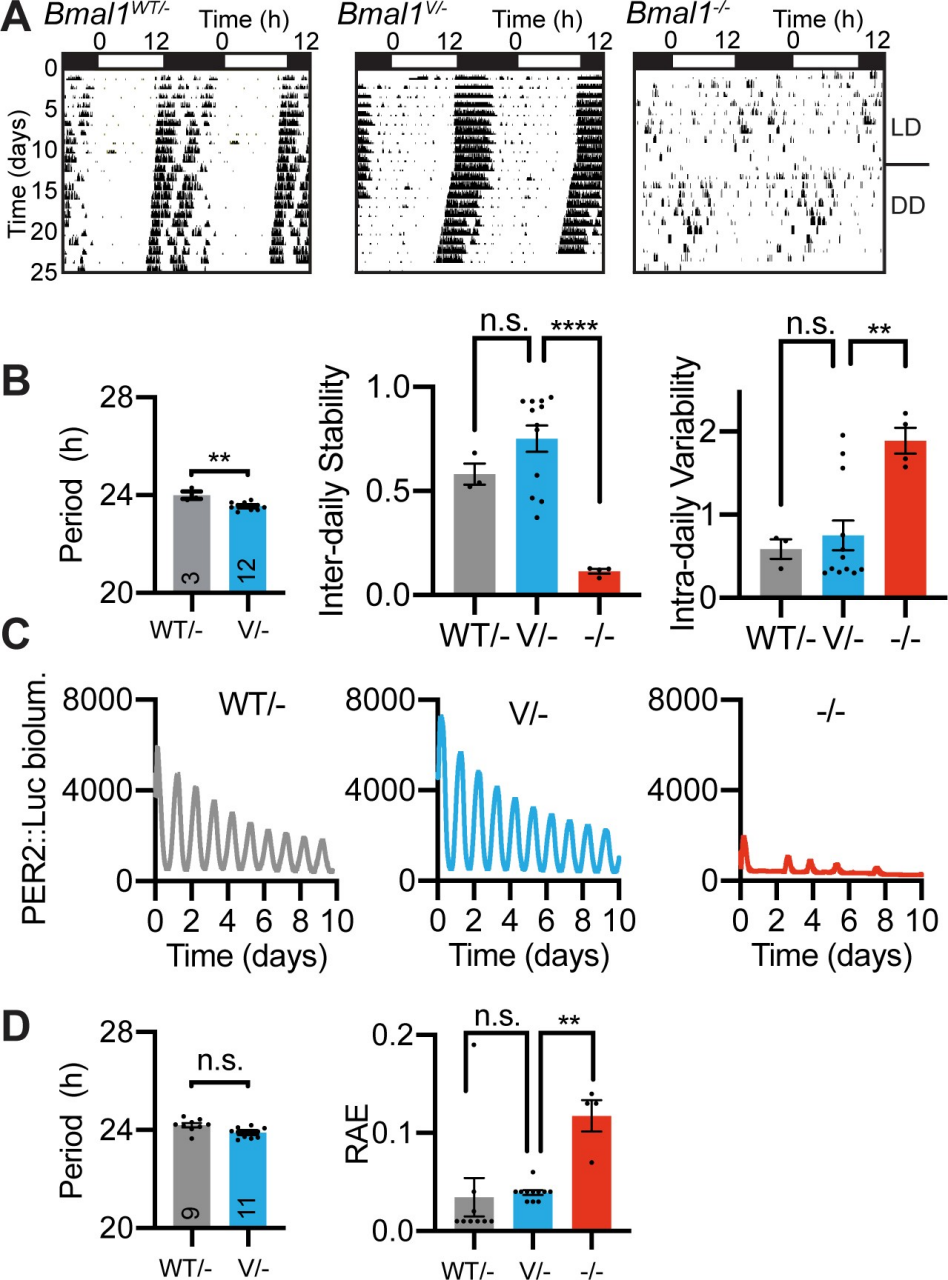
*Bmal1*^Venus^ encodes a Venus::BMAL1 protein with complete circadian functionality. A) Representative double-plotted actograms showing effective rescue of the arrhythmic circadian behaviour of *Bmal1* null (-/-) mice by a single *Bmal1*^*Venus*^ allele (V/-), which is functionally comparable to *Bmal1*^*WT*^ (WT/-). B) Left-to-right: Mean ±SEM circadian periods of wheel-running behaviour, inter-daily stability and intra-daily variability (n ≥3). The latter two are non-parametric robustness measures. C) Representative PER2::Luc bioluminescence traces showing effective rescue of molecular circadian rhythms in SCN slices from *Bmal1* null mice by a single *Bmal1*^*Venus*^ (V/-) allele. D) Mean ±SEM circadian periods of SCN PER2::Luc rhythms and mean ±SEM of RAE robustness measure. Statistics: Ordinary One-way ANOVA for 3 or more groups; 2-tailed, unpaired t-test for comparing 2 groups.

To evaluate the circadian competence of the Venus::BMAL1 protein, we monitored wheel-running behaviour of *Bmal1^WT/WT^, Bmal1^Venus/WT^* and *Bmal1^Venus/Venus^* mice, which all showed well organised rhythms when entrained to light/dark (L/D) cycles or when free-running in constant darkness (DD), with comparable circadian periods (S3A and S3B Fig). We then inter-crossed Venus::BMAL1 and PER2::Luciferase (PER2::Luc) reporter mice to perform bioluminescent recording of molecular circadian rhythms. Organotypic slices of *Bmal1*^*WT*/*Venus*^ and *Bmal1*^*Venus*/*Venus*^ SCN showed clear circadian bioluminescence rhythms, indistinguishable from WT SCN (S3C and S3D Fig). Explant cultures of mammary gland, hip cartilage and IVD from *Bmal1*^*Venus*/*Venus*^ similarly demonstrated clear WT-like circadian rhythms (S3E and S3F Fig). Further, primary MEFs isolated from *Bmal1*^*Venus*/*Venus*^ mice demonstrated robust circadian oscillations from lentivirally transduced *Bmal1-*Luc or *Per2-*Luc circadian transcriptional reporters, confirming the ability of Venus::BMAL1 to drive, respectively, RORE- and E-Box-containing regulatory elements in a cell-autonomous manner (S3G Fig). Moreover, time-course qPCR measures of liver samples from *Bmal1*^*Venus*/*Venus*^ mice showed robust rhythmicity of clock genes *Bmal1*, *Nr1d1* and *Dbp* (S3H Fig), and as a final test of the *Bmal1^Venus^* allele, we crossed it against the *Bmal1^null^* allele. As expected, *Bmal1^null/null^* mice failed to exhibit circadian wheel-running behaviour, whilst SCN slices lacked robust PER2::Luc rhythms (Fig 2). A single copy of the *Bmal1^Venus^* allele was, however, sufficient to rescue both behavioural and SCN PER2::Luc circadian rhythms when paired with a *Bmal1^null^* allele (Fig 2). Thus, in the SCN, peripheral tissues and cells, genomically encoded Venus::BMAL1 is not a dominant negative nor a null mutation. Rather, it is fully functional and sufficient for molecular pacemaking, engaging with core regulatory elements of the TTFL (RORE- and E-boxes) and able to drive circadian behaviour as competently as wild-type BMAL1.

### Visualisation of the dynamic behaviour of Venus::BMAL1 across circadian time and the cell-cycle

**Fig 3:**
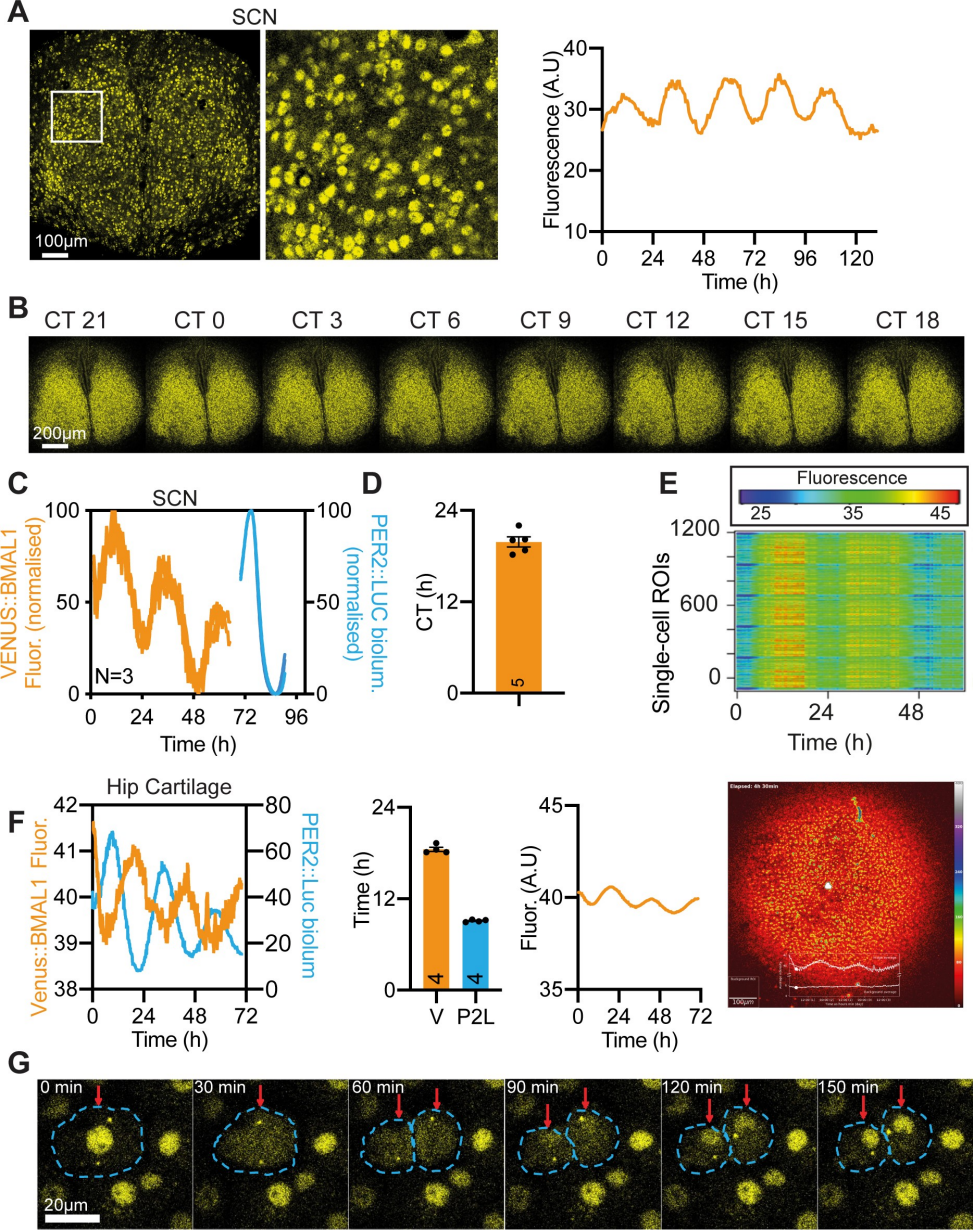
Real-time imaging of the dynamic behaviour of Venus::BMAL1. A) Representative confocal snapshot from live imaging of Venus::BMAL1 in an SCN organotypic slice with associated close-up image (centre, white box), and with background subtracted (BGS) average fluorescence signal over the course of the 6 days of recording (right). B) Montage showing Venus::BMAL1 fluorescence snapshots from a representative SCN slice across the circadian day (with circadian time back-calculated from PER2::Luc rhythms). C, D) Peak fluorescence of Venus::BMAL1 in the SCN was antiphasic to PER2::Luc, with peak mean ±SEM CT of 19.9 ±0.66 hours (PER2::Luc peak set as CT12; n = 5 animals). Normalised, consecutive Venus::BMAL1 and PER2::Luc time-lapse recordings within the same 3 SCN slices used for phase mapping. E) Raster plot showing rhythmic Venus signal across more than 1000 regions of interest across the SCN slice. F) As in (A, C-D) but for hip cartilage tissue explant. Venus intensities were filtered in the pseudo-coloured images to visualise signal fluctuations. Hip cartilage explants from the same Venus::BMAL1/PER2::Luc mouse (opposite limb) were subjected to parallel bioluminescence and fluorescence recording. Results of bioluminescence and fluorescence recordings from left and right hip cartilage explants of the same mouse were plotted together to ease comparison. CT0 corresponds to time of dexamethasone treatment. Despite the difference in amplitude, the antiphase oscillation between Venus::BMAL1 and PER2::Luc was clearly visible (n = 4 animals). G) Montage of Venus::BMAL1 imaging of primary chondrocytes, showing sequential movement of BMAL1 out of the nucleus during mitosis, and fast accumulation in the nuclei of daughter cells. These changes confirm that the Venus::BMAL1 fusion protein is an effective reporter of cytoplasmic localisation of BMAL1, and so the low cytoplasmic levels observed in peripheral cells and SCN are an accurate reflection of the behaviour of endogenous BMAL1.

The *Bmal1^Venus^* allele was created to enable real-time imaging. In living *Bmal1*^*Venus*/*Venus*^ SCN slices, fluorescence was again very strongly nuclear and cytoplasmic signal barely detectable (Fig 3A). Time-lapse confocal imaging showed that nuclear Venus::BMAL1 fluorescence was present at all circadian times (CT) (S3 Movie, Fig 3B), but that it nevertheless exhibited a clearly discernible circadian oscillation (Fig 3A and S4A Fig), albeit of low amplitude (4.6 ±0.9% of baseline; n = 6; S4B Fig). By cross-registering Venus::BMAL1 with PER2::Luc within the same slice (PER2 peaks at circadian time (CT12) (Fig 3C), the peak of Venus::BMAL1 was assigned to the middle of circadian night: CT19.9 ±0.7 (Fig 3D), consistent with the peak of *Bmal1* mRNA expression [5]. Rhythmicity was also evident at the level of individual cells, which showed tight synchronisation of cellular Venus::BMAL1 expression across the SCN (Fig 3B and 3E). These observations contrast with earlier reports of arrhythmic BMAL1 expression in the mouse SCN, based on immunostaining of time-course samples collected from different animals [17]. By serial imaging of the same living SCN, we can reveal the low amplitude oscillation of endogenous BMAL1 expression in the SCN. The circadian expression of Venus::BMAL1 nevertheless contrasted with that of PER2 expression in two critical ways. First, it peaked in circadian night and therefore sits in approximate antiphase to the rhythm of PER2 expression. Second, the amplitude of the Venus::BMAL1 oscillation is much lower than that of PER2 (PER2::Luc amplitude = 166 ±14.6%, PER2::Venus amplitude = 46.8 ±9.6%). Importantly, the fluorescent signal intensity of Venus::BMAL1 did not fall to practically zero, unlike that seen at the nadir of PER2 expression.

To expand these studies to peripheral tissues, simultaneous fluorescent imaging and bioluminescent recording of paired hip cartilage explants (left and right from the same mouse) were used for direct comparison of BMAL1 and PER2 rhythms. Venus::BMAL1 exhibited a clear circadian rhythm (period = 25.64 ±0.59h; n = 4) (Fig 3F, S4A, S4C and S4D Fig) with a low amplitude (6.64 ±0.6%; n = 4) similar to that of the SCN. In contrast the PER2::Luc rhythm of the paired explant had a high amplitude (~250%; n = 4), with a very low nadir (Fig 3F and S4C Fig). The Venus::BMAL1 peak occurred ca. 9.4h later than the PER2::Luc peak in hip cartilage (Fig 3F), comparable to the lag observed in the SCN. To test whether the rhythm of endogenous BMAL1 expression is cell-autonomous, fluorescence was monitored in dispersed cultures of dexamethasone-synchronised primary chondrocytes from *Bmal1*^*Venus*/*Venus*^ mice. Single-cell nucleus-tracking revealed heterogeneity in BMAL1 dynamics (S4E and S4F Fig; S4 Movie). Approximately 45% of cells were arrhythmic and ~10% exhibited ultradian rhythms (S4E and S4F Fig). Nevertheless, the remaining ~45% of cells displayed clear circadian rhythms (periods between 15-30 hours) (S4E and S4F Fig), confirming cell-autonomous control of the circadian oscillation of BMAL1. At this stage, it is unclear whether this low amplitude rhythm is essential for proper circadian control of physiology, not least because constitutive BMAL1 expression can rescue circadian behaviour in *Bmal1^null/null^* mice [6] as well as restore circadian *Per2*-Luc rhythms in *Bmal1*^null/null^ fibroblasts, albeit with a delayed phase. It has, therefore, been proposed that cycling of BMAL1 is not essential for the basic operation of the core circadian clock, but instead may be critical to co-ordinate the proper phase relationship of clock output genes and their dependent physiology [18], consistent with the clear rhythm in BMAL1 promoter occupancy on target genes assessed by ChIPseq [19].

Having examined Venus::BMAL1 dynamics over circadian time in post-mitotic SCN and primary tissues, we then used confocal imaging to monitor its behaviour over a different time scale, during the cell-cycle. Before primary chondrocytes entered mitosis, the fluorescent signal was nuclear, but with dissolution of the nuclear envelope it rapidly (within 6 minutes) distributed throughout the cell (Fig 3G; S5 Movie). Venus::BMAL1 signal progressively accumulated in the daughter cells as mitosis progressed. After ca. 90 minutes nuclear signal re-emerged, alongside cytoplasmic BMAL1, which remained high for a further 30- 40 minutes, before subsiding to a stable low intensity, as seen before cytokinesis (Fig 3G). Thus, in common with many other transcription factors and DNA-binding proteins [20,21], BMAL1 is excluded from condensed chromosomes when regular transcription is suspended during mitosis. This raises interesting questions about how circadian timing carries through cell division. The circadian output rhythms (based on clock gene reporters) are known to persist in dividing cells, and daughter cells resume the rhythms of mother cells after mitosis [22, 23]. However, the circadian cycle length of the immediate next cycle is affected by cell division [16] and it may also be phase-shifted. The transient dissociation of BMAL1 from DNA during cell division observed here may contribute to such a transient phase shift, but it also highlights the potential mobility of BMAL1 between nucleus and cytosol. Indeed, BMAL1 carries nuclear localization (NLS) and nuclear export (NES) signals [24], and when all cytoplasmic signals were repeatedly bleached in living cells, there was a drop of nuclear Venus::BMAL1 signal, as shown by fluorescence loss in photobleaching (FLIP) (S4G and S4H Fig) in both fibroblasts (15.1 ±6.1%, n = 3) and chondrocytes (21.7 ±3.3%, n = 3), indicating that even though Venus::BMAL1 is predominantly nuclear, it does shuttle from nucleus to cytoplasm. Key functions of cytoplasmic BMAL1 include its hetero-dimerisation with CLOCK, which is reported as essential for the phosphorylation and nuclear accumulation of CLOCK, and the proteolysis of CLOCK/BMAL1 heterodimers [8, 9, 25]. Taken together, our live imaging data and FLIP analysis clearly indicate tight spatial and temporal control of BMAL1 localisation during daily and cell-division cycles.

### Quantitative imaging of BMAL1

**Fig 4:**
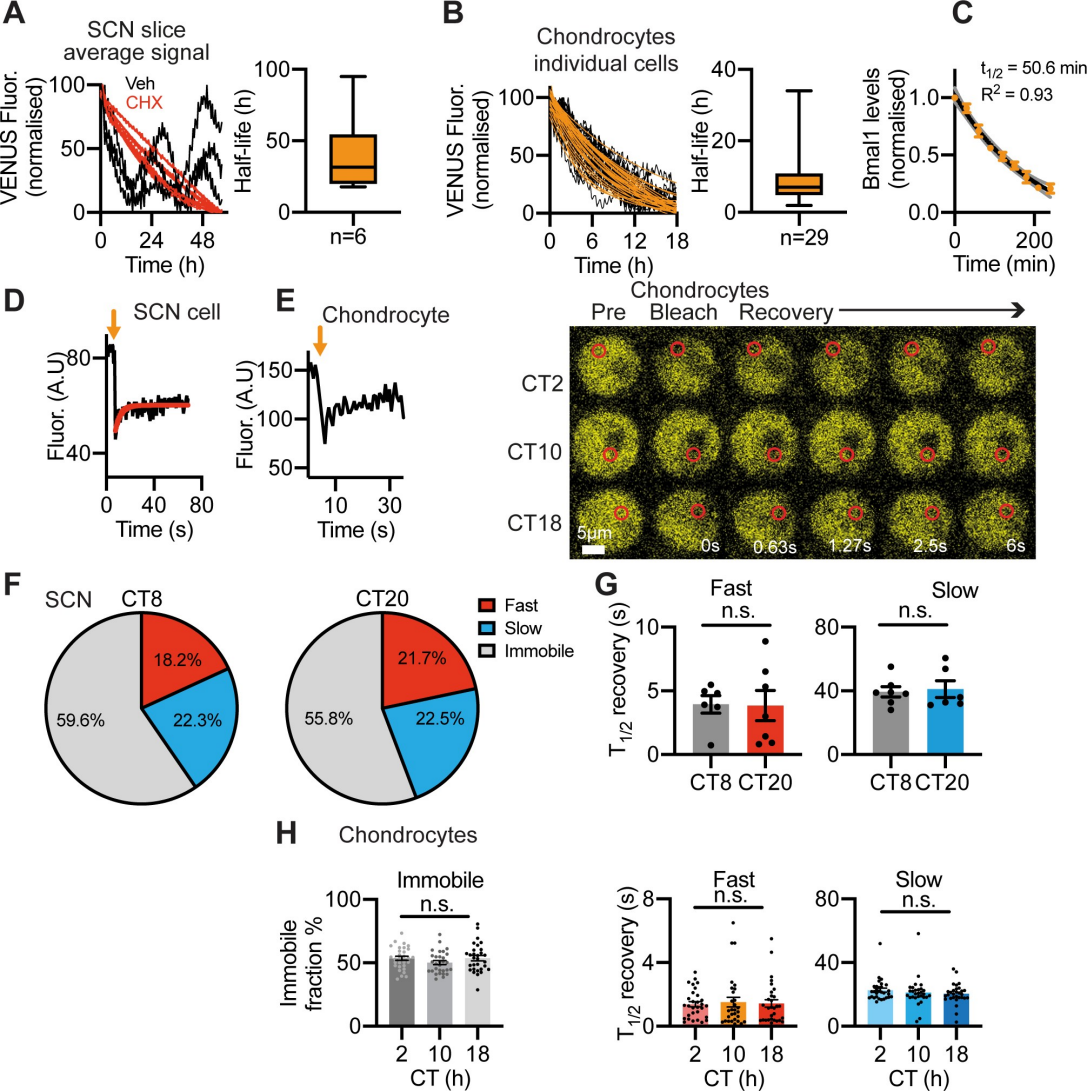
Quantitative analysis of Venus::BMAL1 cellular behaviour. A, B) Half-life of Venus::BMAL1 in SCN and chondrocytes was measured by time-lapse microscopy. A) Organotypic SCN slices were treated with cycloheximide (CHX) (40 μg/mL) to stop *de novo* protein synthesis. Normalised average Venus fluorescence signal from confocal time-lapse recordings of CHX-treated (n = 6 red traces) were compared to vehicle-treated control slices (n = 3 black traces). Note that decline in signal was not complete after 60 hours, thus, half-life measures were estimated from best-fit. Box and whisker plot showing median and interquartile ranges for the half-life of Venus::BMAL1 in SCN slices. B) (Left) Venus::BMAL1 half-life in primary chondrocytes (n = 29 cells). Cells were treated with CHX 5 μg/mL to stop *de novo* protein synthesis. Right) Box and whisker plot showing median and interquartile ranges for the half-life of Venus::BMAL1 in chondrocytes. C) *Bmal1* mRNA stability in primary chondrocytes. Time (min) is following actinomycin D treatment (5 μg/mL). D) Example FRAP experiment showing partial fluorescence recovery after nuclear bleach in the SCN at CT20. E) FRAP analysis in the nucleus of chondrocytes. Left: Fluorescence recovery curve after nuclear bleach. Right: Representative fluorescence images of FRAP experiments in chondrocytes at CT2, 10 and 18. CT0 represents 24 hrs post-dexamethasone. F) FRAP analysis in the nucleus of the SCN revealed two components (fast and slow) of the diffusion kinetics of Venus::BMAL1, as well as an immobile fraction. Pie charts for FRAP analysis of SCN slices at CT8 and CT20 (relative to PER2::Luc rhythms) show percentage of signal explained by fast component, slow component and immobile component in 2-component diffusion model. G) Mobility of Venus::BMAL1 molecules expressed as t_1/2_ of FRAP recovery. Both the fast component (left) and slow component (right) lack a time-of-day effect (mean ±SEM; n = 6). Data were fitted to a 2-component diffusion model, where a fast and a slow component were identified. Note, such high mobility of BMAL1 is in line with other transcription factors in the cell nucleus, but contrasts with the slow kinetics of DNA-bound histones (T_1/2_ in the hours range). H) FRAP analysis in the nuclei of chondrocytes at CT2, 10 and 18. Recovery curves were also fitted to a 2-component diffusion model. Left: % non-recovery signal, i.e. immobile Venus::BMAL1 fraction; middle: fast component; right: slow component (data were calculated based on results from ~30 cells per group, mean ±SEM).

Having confirmed the utility of the Venus fusion as a high-fidelity reporter of endogenous BMAL1 protein, we used it to make quantitative measures of BMAL1 behaviour. The sustained expression of Venus::BMAL1 in all cell types examined contrasts with the strong circadian rhythmicity in *Bmal1* transcription [14, 17, 26]. To test whether BMAL1 protein is more stable in cells than its transcript, we measured the half-life of Venus::BMAL1 in the SCN and peripheral cells by time-lapse microscopy following cycloheximide treatment to inhibit translation. This revealed a long half-life for Venus::BMAL1 of ~31 hours in the SCN and ~8 hours in fibroblasts and chondrocytes (Fig 4A and 4B and S5A Fig). As a comparison, the half-life of *Bmal1* mRNA in cells treated with actinomycin D to stop transcription was 50 min and 140 min in chondrocytes and fibroblasts, respectively (Fig 4C and S5B Fig). This is far less stable than BMAL1 protein and likely accounts for the high baseline, low amplitude oscillation of BMAL1 protein. We previously made similar measures of PER2::Venus half-life of ~2 hours in the SCN [12], consistent with the PER2 half-life of 2-4 hours in SCN, liver and fibroblasts assessed by WB or luciferase recordings [27–31]. These studies also determined CRY1 half-life to be ca. 3-5 hours. The Venus-reported half-life of BMAL1 in the SCN and cells is therefore substantially longer than that of the negative regulators PER and CRY. These findings are consistent with the “Repressilator” design principle of the mammalian circadian clock, which proposes that it is the rhythmic availability of repressors, including the nuclear PER:CRY protein complexes and REV-ERBs, that generates rhythmic gene expression [5, 28, 32–34]. Importantly, according to such a model, there is no absolute requirement for rhythmicity in the abundance of BMAL1 and/or CLOCK to generate rhythmic expression of *Per* and *Cry* [35].

We next used fluorescence recovery after photobleaching (FRAP) to measure the intracellular diffusion kinetics of Venus::BMAL1. Photobleaching a defined area within the nucleus revealed the kinetics of recovery of fluorescence, providing an index of the mobility of BMAL1 and, by inference, its potential association with other proteins or nuclear structures (Fig 4D–4H). FRAP in the SCN revealed three mobility components: fast (ca. 20%), slow (ca. 22%) and immobile (ca. 60%), with respective recovery half-lives of ca. 4s, 40s and absent (Fig 4F and 4G). The fast kinetics of the BMAL1 mobile fraction is consistent with recovery half-times of other nuclear factors (1-14 seconds) [36]. Significantly, the kinetics of BMAL1 and the proportions of the mobile and immobile components did not change with circadian phase (peak and nadir of Venus::BMAL1 signal). In dexamethasone-synchronised primary chondrocytes, FRAP again identified three nuclear components, in proportions and with half-lives similar to those of the SCN (Fig 4H) and, again, mobility of BMAL1 did not change with circadian phase.

**Fig 5:**
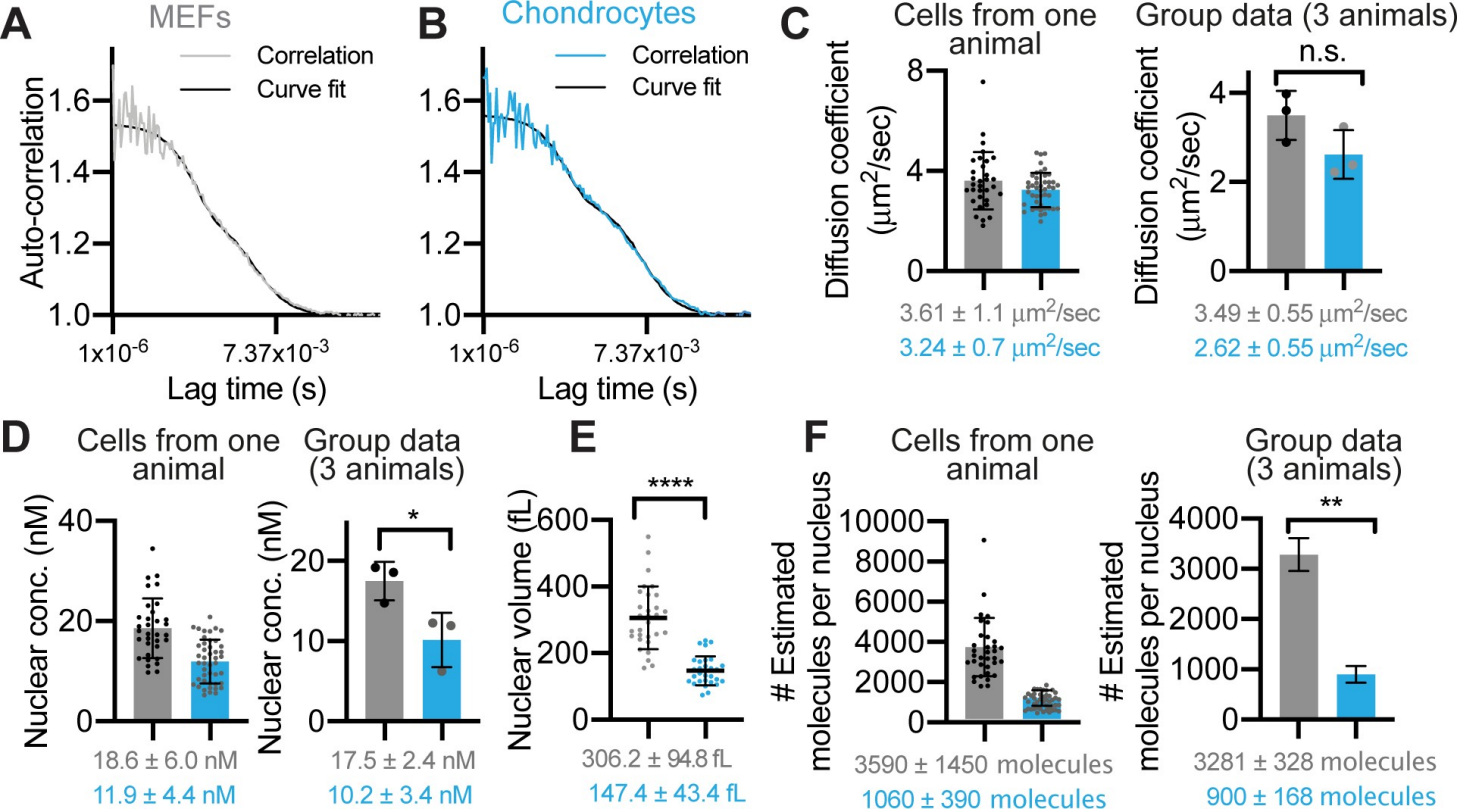
Quantitative analysis of molecular behaviour of Venus::BMAL1 measured by FCS. A, B), FCS of Venus::BMAL1 in unsynchronised MEFs (grey) and chondrocytes (blue). Representative autocorrelation curves were fitted to a one-component diffusion model with a triplet state. C, D) Quantitative analysis of Venus::BMAL1 FCS data. Scatter plots presented in pairs, first, as measurements of cells taken from an individual representative animal, followed by group data: mean averages across three different animals. Mean ±SD; n = 40 cells per mouse, from 3 mice. C) Diffusion coefficient (3.49 ±0.55 μm^2^/s in MEFs and 2.62 ±0.55 μm^2^/s in chondrocytes). D) Molecular concentration (17.5 ±2.47 nM in MEFs and 10.2 ±3.45 nM in chondrocytes). E) Mean ±SD nuclear volume measurements for MEF cells (grey, 306 ±95 fL, n = 30 cells) and chondrocytes (blue, 147 ±43 fL, n = 30 cells). F) Estimated number of Venus::BMAL1 molecules per nucleus in MEFs and chondrocytes. The calculation of the mean was based on the molecular concentration of Venus::BMAL1 in each cell and the average nuclear volume for that cell type, as calculated in E. In the group data (right), the SD was adjusted by using a formula: $\sqrt{{\bar{x}}_{A}^{2}({SD}_{B}^{2})+{\bar{x}}_{B}^{2}({SD}_{A}^{2})}$ to correct for the propagation or error. Statistics: Nuclear volumes: Unpaired two- tailed t-test; concentration, diffusion coefficient and molecular number: Paired two-tailed t-test.

We then performed absolute quantification of the nuclear concentration and mobility of endogenous BMAL1 molecules in live cells under physiological conditions, using fluorescence correlation spectroscopy (FCS) (Fig 5). The FCS data fitted well to a one-component (fast), triplet state diffusion model, as shown by the low level of deviation between the correlation and curve-fit (Fig 5A and 5B and S6 Fig). This yielded a diffusion coefficient of 3.5 ±0.6 μm^2^/s in MEFs and 2.6 ±0.6 μm^2^/s in chondrocytes (mean ±SD of ~40 cells per animal, n = 3) (Fig 5C). FCS analysis in unsynchronized MEFs and chondrocytes revealed the mean concentration of mobile Venus::BMAL1 to be 17.5 ±2.4 nM and 10.2 ±3.4 nM, respectively (Fig 5D). The volumes of cell nuclei (Fig 5E) were measured in order to estimate the absolute numbers of molecules present per cell, revealing 3281 ±328 molecules in MEFs and 900 ±168 molecules in the smaller chondrocytes (Fig 5F). Our measurement of Venus::BMAL1 copy numbers per cell in fibroblasts is likely an underestimate because FCS only measures mobile molecule species. Indeed, a previous report using mass spec-based quantification of BMAL1 estimated ~20,000 copies of BMAL1 per cell, albeit in a different tissue type (mouse liver tissue lysates) [37]. Interestingly, they also showed that the copy number in cells only demonstrated a mild oscillation (with a ~18% amplitude) within 24 hours, consistent with our observation [37]. Taken together, these results indicate that Venus::BMAL1 molecules are highly mobile: they exhibit a single fast diffusion mode under physiological conditions (in FCS) and recover rapidly (within seconds) following bleaching (in FRAP). These properties contrast with PER2 kinetics, which displayed two components (fast and slow) in FCS and a slower recovery after photobleaching [12]. The fast BMAL1 kinetics are consistent with earlier reports that BMAL1-CLOCK occupancy on arrays of *Dbp* E-box repeats can be extremely unstable and dynamic when monitored by fluorescence microscopy [38]. Finally, our data support a mathematical model of the mammalian molecular circadian clocks [39] in two ways: first, neither BMAL1 nor CLOCK are required to oscillate strongly in order to drive a 24-hour rhythmicity. Rather, strong rhythmicity for PERs and CRYs is necessary. Second, the predicted (modelled) circadian rhythm in BMAL1 concentration only fluctuates between 7.2-7.8 nM [38], close to our direct measurements of 10-17 nM. Importantly, low-amplitude oscillation of BMAL1 sits in approximate antiphase to the negative regulator PER2. It is therefore interesting to consider the implications of the changing relative abundance of PER2 and BMAL1 at different circadian phases.

### Conclusion

The Venus::BMAL1 mouse enabled direct quantitative measurement of the spatiotemporal distribution, daily dynamics, intracellular kinetics, half-life and molecular concentration of the master clock protein BMAL1 in an endogenous setting. Our results revealed a low amplitude yet clearly discernible circadian oscillation of BMAL1 with a high mobility within the nucleus. We also show striking contrasts in the stability and diffusion kinetics of BMAL1 (positive regulator) as compared to PER2 (negative regulator); and these contrasting properties may be pivotal to effective operation of the cell-autonomous circadian timing mechanism. More generally, they reveal a complex and intricate regulation at the heart of the circadian molecular oscillator.

## Materials and methods

### Ethics statement

Animal studies were performed in accordance with the 1986 UK Home Office Animal Procedures Act. Approval was provided by the Animal Welfare Ethical Review Board (AWERB) of the University of Manchester (approval no. 50/2506) and of MRC Laboratory of Molecular Biology, Cambridge.

### Animal maintenance and breeding

Animals were maintained at 20°C to 22°C, humidity of 40-50%, under an LD schedule (light on at 7 am; light off at 7 pm), with ad libitum access to water and standard rodent chow. Venus::BMAL1 mice were crossed with PER2::Luc mice [40] for SCN luciferase recordings. They were also crossed into a *Bmal1* null allele mouse, for testing sufficiency of one copy of Venus::BMAL1 to drive behavioural and SCN circadian rhythms. The BMAL1 null was generated by crossing Nestin-Cre mice [30] with Floxed-*Bmal1* mice [41]. PER2::Venus mice were previously described in [12]. Cartilage specific Col2a1-Cre/BMAL1 KO mice were described in [14].

### Design and Generation of CRISPR reagents, microinjection, and identification of positive offspring

In order to create a VENUS::BMAL1 fusion protein expressing mouse line we used CRISPR Cas9. sgRNA targeting the start codon of the *Bmal1* (*Arntl*) gene were identified (sequences TTCTCTGGTCCGCCATTGGA-*AGG* and TCCAATGGCGGACCAGAGAA-*TGG* with the PAM indicated in italics) and sgRNA generated following the method published by Shen et al [37]. Briefly, complementary oligos for the sgRNA target sequences were synthesised, annealed and ligated into pUC57-sgRNA expression vector (Addgene #51132). Following the sequence confirmation plasmids were mini-prepped, and linearised with DraI restriction enzyme, they were used as template in an *in vitro* HiScribe T7 transcription reaction (NEB). RNA samples were purified using Ambion MEGAclear transcription clean up kit and concentration quantified, aliquoted and frozen at -80^o^C ready for microinjection.

For the HDR repair template we synthesised a pUC57 vector (Genscript) with the 800 bp immediately 5’ and 3’ to the ATG up and downstream of the VENUS fluorescent protein sequence with a double flexible linker [42]. 3 bases in the 3’ homology arm were synonymously altered (i.e. same amino acid produced from different codon) to prevent the potential for CRISPR re-cutting the desired HDR allele. We generated a linear DNA repair template by amplifying the 2.4 kb Homology-Venus-Linker-Linker-Homology with Phusion polymerase (NEB). PCR product was gel purified from Ultrapure agarose gel (Monarch, NEB) before being subjected to a further round of column purification (Monarch PCR clean, NEB), and eluted in RNase free injection buffer (TrisHCl 1mM, pH 7.5, EDTA 0.1mM), concentration quantified and stored at -20^o^C.

For injection each *in vitro* transcribed sgRNA was combined with Cas9 recombinant protein and incubated at room temperature for 10 min, and combined with the purified DNA (final concentrations; sgRNA 50 ng/μl, Cas9 protein 100 ng/μl, donor DNA 10 ng/μl). The injection mix was pronuclear microinjected into one-day single cell mouse embryos. Zygotes were cultured overnight and the resulting 2 cell embryos surgically implanted into the oviduct of day 0.5 post-coitum pseudopregnant mice.

Pups were initially genotyped for presence of the *Venus* gene using primers QJM01 Geno F1 catgacctcagtgccacaac and QJM01 VenusR1 gtcttgtagttgccgtcgtc. 9/35 mice were positive for this reaction, and were taken forward for genotyping for correct insertion by PCR using primers flanking the homology arms in combination with Venus cds specific primers (5’ reaction primer sequences QJM01 GenoF3 tctttaattatgcaagcggatgct and QJM01 Venus R3 acttgtggccgtttacgtcg, 3’ reaction primer sequences QJM01 VenusF1 cgggatcactctcggcatgg and QJM01 GenoR2 ctgcctcagtgaaacgaaacc). Mouse 3 and mouse 4, which generated positive PCR products for both reactions, were Sanger sequenced and results aligned to predicted allele sequence. A colony was established from founder 3 and heterozygote and homozygote pups bred and genotyped using the primers Het/HomF agtgggagcctcattcttcc and Het/HomR gtcccaaggctcttactggt, and western blots used to confirm fusion protein size shift.

### Analysis of circadian behaviour

For Manchester animal cohort: Ten-week-old mice (*Bmal1^WT/WT^, Bmal1^WT/Venus^, or Bmal1^Venus/Venus^* from littermates) were housed in individual cages in light-tight cabinets (Tecniplast), equipped with disposable cardboard running wheels, as described previously [30]. Activity was recorded by a chronobiology kit in 10-minute time bins (Stanford Software Systems). The mice were maintained at LD cycles (light on at 7 am; light off at 7 pm) for two weeks. Settings of the light-tight cabinets were then changed to constant darkness, for another two weeks. ClockLab (Actimetrics) was used to generate double-plotted actograms and perform χ^2^ periodogram analyses.

For MRC Laboratory of Molecular Biology animal cohort: Eight to ten week old mice were housed in individual cages in light-tight cabinets (Tecniplast), equipped with activity mouse wheel cages (Actimetrics). Activity was recorded by ClockLab data collection software in 6-minute bins (Actimetrics). The mice were maintained at LD cycles (light on at 7 am; light off at 7 pm) for two weeks. Activity profiles were generated using ClockLab (Actimetrics) and used to apply Non-Parametric Circadian Rhythm Analysis (NPCRA) to 10 circadian days of wheel-running data, as described previously [3], to calculate: Intra-daily Variability (IV): Non-parametric frequency of activity-rest transitions within a day, with a range of between 0-2 E.g. A Sine wave would have a value of 0 and Gaussian noise would have a value of 2. Inter-daily Stability (IS): Matching of activity patterns on day-to-day basis, ranging from 0 (Gaussian noise) to 1 (high-stability). Robust behavioural activity is characterised by low IV and high IS. ClockLab (Actimetrics) was used to generate double-plotted actograms with onsets of activity and phase angle of entrainment was calculated from 10 days of wheel-running data measuring the difference in time of the point in the entraining cycle (lights on) against the onset of activity.

### Western Blotting

Mouse lung tissues were lysed in protein sample buffer (50 mM Tris-Hcl, 10% glycerol, 2% SDS, 100 mM DTT, 5% β-mercaptoethanol and bromophenol blue to colour, pH 6.8) with cOmplete EDTA-free protease inhibitor cocktail (Merck). Protein samples were quantified using Bradford protein assay, and then electrophoresed on 4-20% Mini-PROTEAN TGX Precast Protein Gels (Bio-Rad). Subsequently, gels were transferred to nitrocellulose membranes, then blocking step was conducted in Odyssey Blocking Buffer (LI-COR) for one hour, followed by incubation with primary antibodies (either anti-BMAL1, Cell Signaling; or monoclonal anti-α-Tubulin, Merck) overnight at 4°C. Secondary antibodies IRDye 680/800 were used and protein signals were analysed using LI-COR Odyssey CLx.

### Organotypic SCN slice preparation

SCN slice cultures were prepared as previously described [12] and imaged after 2-3 days after preparation for confocal imaging, or kept for 7 days in culture prior to bioluminescence recording.

### Real-time fluorescence imaging of peripheral tissues and cells

Real-time recording of VENUS signals was performed using a Zeiss confocal microscopy (either LSM780 or LSM880) in a humidified CO_2_ incubator (at 37°C, 5% CO_2_). Either cells or tissues were cultured in HEPES and sodium bicarbonate-buffered recording medium on 35-mm glass bottom CELLview dishes (Greiner Bio-one). For tissue imaging, Millicell 0.4 μm cell culture inserts (Merck) were used. Images were captured using either a 10x or 40x objective, with excitation by 514 nm laser light. The emission was collected between 518 and 535 nm, with a pinhole set to one Airy unit. To identify the localisation of Venus signals, either DRAQ5 or DAPI was applied to counterstain nuclei. Data capture was conducted using ZEN2010 software (Zeiss). Images were individually band-pass filtered (A trous wavelet) to remove both high frequency noise and stationary background. After pre-filtering the timelapse images, Imaris was used to track the nuclei over time using the "spots tracking" function. Mean brightness along the track and at each time was collected for further analysis. Next, periodicity was determined by FFT power spectrum analysis and autocorrelation (negative at 12 hours, positive at 24 hours). The power spectral density was calculated for each track after filling missing data (forward fill function) and suitable detrending. For each of the resulting spectra, the frequency of the maximum peak was collected. The histogram of these frequencies was plotted. The cells were split into 3 groups: those that exhibit a periodic component somewhere between 15 and 30 hours (circadian), those whose periodicity was below 15 hours (ultradian) and those that were greater than 30 hours and inferred as being arrhythmic.

### Imaging of the SCN

Fixed tissue: Images of fixed sections were acquired using Zeiss 710 inverted confocal system (Zeiss, Germany) using 63x objective and tile-scanned to cover the entire SCN.

Time lapse imaging: Fluorescence timelapse recordings of Venus::BMAL1 in SCN organotypic slices were acquired using Zeiss LSM780/880 inverted confocal system (Zeiss, Germany), and maintained at 37°C. Samples were placed in air-tight glass-bottom dishes (Mattek, USA). Images were acquired using 10x objective, 30 seconds scan time per frame, 2 frames per hour, for 6 days for longer time lapse or 60-70 hours for shorter time lapse. Average traces from time lapse experiments used full frame averages. Both raw data, background subtracted and normalised traces are shown in the figures. Background was subtracted from an ROI outside of the SCN using the “Background Subtract from ROI” plugin in ImageJ (NIH, USA). Amplitude measures were calculated as the baseline-to-peak, as a percentage of the baseline intensity.

### Immunohistochemistry

As described previously, immunohistochemistry was performed using the DAB staining method [14, 15]. Briefly, paraffin embedded mouse tissues (either hip cartilage or intervertebral discs) were sectioned, deparaffinized and rehydrated. Following antigen retrieval using Trypsin (Merck), tissue slides were blocked in goat serum for one hour, and then incubated with primary antibody in blocking buffer overnight at 4°C. Anti-BMAL1 was raised against the C-terminal 15 residues of mBMAL1 (GLGGPVDFSDLPWPL) [43]. Secondary antibody goat anti-rabbit IgG (Vector Laboratories) was used, and methyl green was applied as a counter stain for DAB. One of the sections on each slide was used as a no-primary antibody control to establish the background level.

### Immunofluorescence

For the SCN, mice were sacrificed at ZT 9 (lights off for animals is ZT12), and brains post-fixed in 4% PFA (in 0.01M phosphate buffer). Immunostaining protocol carried out as previously described [12]. Primary antisera: rabbit anti-AVP (1:1000; Bachem, USA), rabbit anti-VIP (1:1000, Immunostar, USA), rabbit anti-GRP (1:1000; Immunostar, USA), rabbit anti-BMAL1 (1:500; generated in-house) [43]. Secondary antiserum: Alexa 647(1:500, Life Technologies, USA). Sections mounted with Vectashield containing DAPI (Vector Labs, USA). For cartilage, immunostaining protocol carried out as previously described [14]. Primary antiserum: Anti-BMAL1 [43]. Secondary antiserum, Alexa Fluor 647 (Abcam, UK). Sections mounted with Vectashield containing DAPI (Vector Labs, USA).

### Bioluminescence recording

Tissue explants or cells were prepared as described before [12, 14, 15, 30, 40, 44]. Pre-warmed HEPES and sodium bicarbonate-buffered recording medium was applied for the bioluminescence recording.

For peripheral tissues and MEFs, the signals were recorded in real-time, using a Lumicycle apparatus (Actimetrics). A 24-hour moving average algorithm was used to subtract baseline during analysis.

For SCN organotypic slices whole-slice bioluminescence emissions were detected by photon multiplier tubes (Hamamatsu, Japan) maintained at 37°C and set-up to count emitted photons every second in 6 minute bins. Bioluminescence rhythm data was analysed using a non-linear fast-Fourier Transform (NL-FFT) within the BioDare software package: https://biodare2.ed.ac.uk (Prof. A. Millar, University of Edinburgh) [45]. Circadian period, amplitude, relative amplitude error (RAE, a measure of rhythm robustness), were calculated for each dataset.

### TaqMan Gene Expression Assays

Mouse liver samples were harvest every 4 hours for 24 hours. As described before [44], RNA was extracted using the RNeasy Mini Kit (Qiagen), followed by cDNA preparation with a High-Capacity RNA-to-cDNA Kit (Thermo Fisher Scientific)‎. Gene expression was analysed using quantitative real-time PCR, with TaqMan probes, including *Bmal1/Arntl*: Mm00500226_m1; *Nr1d1/Rev-erba*: Mm00520708_m1 and *Dbp*: Mm01194021_m1. Results were normalised to the values for *Gapdh* expression, using the 2^-ΔΔCt^ method.

### Lentiviral transduction

The lentiviral transduction was performed using our previous published method [15]. Briefly, lentiviral particles containing either *Bmal1*-Luc or *Per2*-Luc reporter were produced in HEK 293T packaging cells, and then transduced to primary mouse fibroblasts. Cells were synchronized with dexamethasone before lumicycle recording.

### Isolation of mouse primary articular chondrocytes

Femoral head tissues from 5-day-old mice were harvested and digested in 3 mg/mL collagenase D (in Hank's Balanced Salt Solution, H9394 Merck) for 45 mins at 37 °C, to remove all the soft tissues. Following agitating tissue fragments for 30 seconds by pipetting, the pieces were incubated in 0.5 mg/mL collagenase D overnight at 37 °C. Individual cells were collected through 40μm cell strainers, and then pelleted by centrifugation for 10 min at 200g. Cells were cultured in Dulbecco’s Modified Eagle’s Medium (D6429, Merck) supplemented with 10% FBS, 4 mM L-glutamine, 1% NEAA and antibiotics before imaging [46].

### Isolation of mouse embryo fibroblasts (MEFs)

Mouse embryo fibroblasts were isolated on embryonic day 13.5 (E13.5). All fetuses were harvested by removing head, liver and heart. Ice-cold 0.25% trypsin-EDTA media (3 ml per embryo) was used to digest tissues overnight at 4 degree. Following further incubation in a 37 degree water bath for 30mins, the digested tissues were broken into cell suspension by vigorously pipetting [47]. Cells were plated in T75 tissue culture flasks, in DMEM medium (D6429, Merck) with 10% FBS and antibiotics before imaging.

### Isolation of mouse intervertebral disc (IVD) cells

Mouse primary IVD cells (AF) were isolated from tail spines of 1 month old mice. Tendons were removed from the spine then IVDs were dissected out. A sharp blade was used to separate AF rings from CEP and the NP structure was removed under a dissection microscope. AF tissues were minced thoroughly prior to digestion. Minced tissues were pre-digested with 0.1% Pronase in DMEM F12 + 1% P/S for 30 minutes, with frequent agitation. Tissues were subsequently digested in 0.3% Collagenase II in DMEM F12 with 10% FBS and 1% P/S for 90 minutes. This was followed by washes and culture in DMEM F12 with 10% FBS and 1% P/S.

### Half-life analysis

To detect the degradation of Venus::BMAL1, either primary fibroblasts or chondrocytes were treated with 5 μg/ml cycloheximide (Merck). Fluorescence was recorded every 6 mins for 20 hours, using the Zeiss LSM880 system with a 40x objective. For the SCN, organotypic slices were treated with 40 μg/ml cycloheximide (Sigma Aldrich, USA) or vehicle and recorded from every 30 mins for 70 hours using the Zeiss LSM710 system with a 10x objective. The analysis of BMAL1 half-life was performed in Prism, and data are shown as median with error bars showing the range (GraphPad Software). The data were normalised to the smallest and largest fluorescence intensity values for each sample. The normalised data were fit to a one-phase decay (Y = (Y0 - Plateau)*exp(-K*X) + Plateau), and half-life derived from this curve fit. As can be seen by the robust Venus::BMAL1 fluorescence oscillations, vehicle treated SCN slices did not exhibit acquisition bleaching, thus bleaching correction was not applied in cycloheximide treated slices.

Actinomycin D (Sigma Aldrich, USA) was used to block de novo mRNA synthesis and study *Bmal1* mRNA decay in mouse primary fibroblasts and chondrocytes. Actinomycin was added to the culture media at a final concentration of 5 μg/mL (0.05% DMSO). Cells were harvested for RNA isolation at 0, 30, 60, 90, 120, 150, 180, 210 and 240 min after treatment. Total RNA was isolated using the PureLink RNA Mini Kit (Thermo Fisher) as per manufacturer’s instructions. Complementary DNA (cDNA) was generated from 400 ng of total RNA using the high capacity RNA-to-cDNA kit (Applied Biosystems) according to the manufacturer’s instructions. RT-qPCR was performed in a total volume of 10 μL, comprising 5 μL of Takyon MasterMix (Eurogentec), 0.5 μL of TaqMan gene expression assay probes, 1 μL cDNA template at a concentration of 10 ng/μL and 3.5 μL of nuclease-free water (Sigma). No template controls were included for all reactions. RT-qPCR was carried out on a StepOne Real-Time PCR System (Applied Biosystems) according to the following programme: 2 min at 50 °C to prevent carryover, 3 min at 95 °C to activate the Takyon enzyme, and 40 cycles of denaturation at 95 °C for 30 sec and annealing/extension at 60 °C for 60 sec. Gene expression data was obtained using the 2^-ΔΔCt^ method in which samples were first normalised to the mean levels of the reference genes *Gapdh* and *Rpl13a*; and relative normalisation was performed in relation to time 0. To estimate the mRNA half-life of *Bmal1*, data was fitted to a one-phase exponential decay model (least squares fit) using GraphPad Prism version 8.2.0. TaqMan gene expression assay probes used in the present study: *Bmal1*, Mm00500226_m1; *Gapdh*, Mm99999915_g1, *Rpl13a*, Mm05910660_g1.

### Fluorescence recovery After Photobleaching (FRAP)

To detect the mobility of BMAL1 protein, fluorescence recovery after photobleaching was applied using either the Zeiss inverted confocal system (Zeiss, Germany)- LSM780 for SCN slices with 40x water immersion objective, or LSM880 system for peripheral tissues with a 63x oil objective. The experiments were performed at three time points (CT2, CT10, CT18) in peripheral tissues and at either CT8 or CT20 (predicted peak and through respectively) in SCN slices, as assessed by PER2::Luc rhythms. Only the area around the selected bleaching ROI was imaged to minimise acquisition bleaching. For each photobleaching, 10 consecutive pre-bleach images were acquired at low laser intensity. Following the photobleaching with total laser intensity, post-bleach images were acquired at 10 frames per second. For the SCN, 10 frame pre-bleach acquisition at 10% laser power, 3 μm diameter discs bleach in the nucleus at 100% laser power, followed by post bleach acquisition every 0.5 seconds. In parallel, for each measurement, background ROI, and unbleached ROI were also monitored, to correct for background and acquisition bleaching. The T_1/2_ was calculated within the Zeiss Zen software, based on a selected diffusion model (best-fit was 2-component, passive diffusion). The software was also used to calculate the percentages for the proportion of fluorescence recovery signal that could be explained by components 1, 2 or immobile fractions. The immobile fraction cannot be differentiated from very slow moving molecules.

### Fluorescence Loss in Photobleaching (FLIP)

FLIP images were gathered on a Zeiss LSM880 system using a 40x oil objective. Using Zen 2010 software, bleach area was defined by drawing an ROI around the cytoplasm of the cell of interest, excluding the nucleus. 5 consecutive baseline images were captured prior to 50 iterations of cytoplasmic bleaching at full laser power of 514 nm for ~15-30 seconds, dependent on cytoplasmic area. Images were then captured every 3.73 seconds following bleaching. Data were processed by subtracting background noise and normalised to the nuclei of adjacent non-bleached cells, to account for the long bleach duration which contributes to an observed gradual photobleaching of all cells within the field of view over the course of the experiment.

### Fluorescence Correlation Spectroscopy (FCS)

FCS measurements were conducted using a Zeiss LSM880 system and plan-apochromat 63x NA 1.4 objective as previously described [12]. Briefly, Venus fluorescence was excited by 514 nm wavelength at 1% laser power to yield a minimum of 1kHz counts per molecule while avoiding photobleaching. Fluorescence emission was collected between 518 and 535 nm with the pinhole set to one Airy unit. 5 runs of 5 seconds each were conducted per cell. Data were collected and correlation curves fit to raw data using Zen 2010 software.

### Spectral imaging and linear unmixing

Primary chondrocytes and embryonic fibroblasts were cultured in HEPES and sodium-bicarbonate-buffered medium on 35-mm glass bottom dishes in a humidified incubator at 37 °C and 5% CO_2_. Cells were stained with the CellMask Deep Red Plasma membrane stain (C10046, Invitrogen) prior to imaging, according to the manufacturer’s instructions. Cells were excited with the 514 nm and 594 nm laser lines using the Lambda stack acquisition mode of the Zeiss LSM880 multiphoton microscope. Images were captured using a 63× 1.46 Korr M27 objective with immersion oil. Emission light was collected at wavelengths ranging from 521 nm to 690 nm. Regions of interest (ROI) corresponding to the Venus::BMAL1 (yellow), CellMask Deep Red Plasma membrane stain (red) and auto-fluorescence (blue) signals were selected using the Zeiss 2.1 software (Zeiss). Subsequently, the linear unmixing function was used to calculate the contribution of each fluorophore in the acquired Lambda stacks. 3D reconstructions of the linearly unmixed z-stacks were performed using the Imaris software.

Cell volume measurements were acquired by Z-stack imaging of Venus::BMAL1 nuclear fluorescence in trypsinised cells (n = 30 cells). Using a Zeiss LSM880 confocal microscope with 63x objective, fluorescence was excited at the 514 nm laser line (15% laser power) and emission collected from 516-562 nm. Z-stack images were visualised in 3D using Imaris (Bitplane Inc.) version 7.4.0 and cell volume quantified with the use of the Surfaces tool.

### Data analysis

Data were evaluated using Student’s t-test, Mann-Whitney U-test, or one-way ANOVA with Tukey test as indicated. A *P* value of less than 0.05 was considered statistically significant. Results were from at least three independent experiments, and presented as the mean ± SEM or mean ± SD. Graphs and statistics were generated using Prism (Graphpad, USA), unless specified. Image analyses were made using ImageJ (NIH, USA). Semi-automated cell counts were made using the “Nucleus Counter” plug-in within ImageJ. Percentage Venus::BMAL1 colocalization with neuropeptides was calculated using the Nucleus Counter applied to a new channel only containing signal if present in both Venus and neuropeptide channels compared to the counts in the neuropeptide image channel. Mander’s co-localisation analysis was conducted using the Mander’s Coefficient plug-in within ImageJ. Average traces from time lapse experiments used full frame averages. Both raw data and normalised traces are shown in the figures. Raster plots were generated from SARFIA package [48] within the Igor Pro (Wavemetrics, USA). Single cell-like regions of interests were assigned using wavelet-based edge detection.

## Supporting information

S1 Fig Generation and validation of Venus::BMAL1 mouse. A) PCR analysis of 5’ (1050bp) and 3’ (970bp) genome-transgene integration. The number above each band denotes mouse ID. B) DNA sequencing to confirm the “bridge” junctions of the Venus-*Bmal1* allele. All junctions were correct as predicted. C) PCR genotyping of breeding colonies indicating band patterns for wild type (WT), heterozygotes (Het) and homozygotes (Hom). WT allele - 297bp; Knock-in allele - 1096bp. D) Validation of BMAL1 antiserum in knee and hip femoral head cartilage from WT and Col2a1-Cre/*Bmal1*^*-/-*^ KO mice. (Left panel) Immunohistochemistry: anti-BMAL1 shown in brown. (Right panels) Immunofluorescence: anti-BMAL1 shown in red in merged image and white in single channel image. Related to Fig 1C.
(TIF)

S2 Fig BMAL1::Venus localisation in the SCN and peripheral tissues. A) Low magnification of Venus::BMAL1 (green) co-localisation with neuropeptide-ir (red) in the SCN, co-stained with DAPI (blue). Scale bar = 100μm. Note that this is a composite image from 5x5 tile scan resulting in minor image-join artefacts (in relation to Fig 1E). B) SCN cell-type composition was determined by cell counts of different neuropeptide-expressing cells (n = 4-5 mice). Note there was no difference in the cellular composition between Venus and non-Venus mice. C) Left: representative confocal micrograph showing Venus::BMAL1 fluorescence in hip cartilage tissue explant. Right: a merged image of Venus and DRAQ5 shows nuclear localisation of Venus::BMAL1 in hip chondrocytes.
(TIF)

S3 Fig *Bmal1*^Venus^ encodes a Venus::BMAL1 protein with complete circadian functionality. A, B) Representative double-plotted actograms showing normal wheel-running behaviour of wild type (WT/WT or WT), heterozygous (WT/V or Het) and homozygous (V/V or Hom) mice. Circadian periods for wheel-running during 12:12 light-dark (LD) cycling conditions and subsequent continuous darkness (DD) (n ≥4). There were no significant differences in phase of entrainment to the L/D cycle, the robustness of circadian behaviour (intra-daily variability and inter-daily stability non-parametric analyses), nor the duration of the main activity bout (alpha) (n >4; mean ±SEM). C) Representative PER2::Luc molecular rhythms in SCN organotypic slices. D) Mean ±SEM circadian periods for the SCN (n >18 for each group). E) Representative PER2::Luc rhythms in peripheral tissue slices (IVD, mammary gland and hip cartilage) of WT (black) and Venus::BMAL1 (orange) mice. F) Mean ±SEM circadian periods for peripheral tissues. G) Fibroblasts from Venus::BMAL1 mouse demonstrate normal circadian pacemaking as shown by antiphase oscillations of RORE- and E-Box-containing clock reporters. *Bmal1*-Luc or *Per2*-Luc reporters were transduced into primary Venus::BMAL1 MEFs by lentivirus. Cells were synchronised by dexamethasone before bioluminescence recording. H) Robust rhythmicity of endogenous clock genes in liver of Venus::BMAL1 mouse determined by qPCR.
(TIF)

S4 Fig Real-time imaging of the dynamic behaviour of Venus::BMAL1. A) Average fluorescence recordings related to Fig 3A (left) and Fig 3F (right). Raw fluorescence intensities shown in orange and background fluorescence shown in black/grey. Background intensities are subtracted from raw intensities and shown as background subtracted, in the main figure. B) (Left) Representative raw traces from SCN timelapse recordings: PER2::Luc (grey), PER2::Venus (blue) and Venus::BMAL1 (orange) plotted on the same graph to compare their amplitudes of circadian oscillation. (Right) Group data (mean ± SEM) showing percentage amplitude (relative to baseline) for each reporter. Note that PER2::Venus and Venus::BMAL1 use the same fluorescence reporter so are most comparable in terms of assessing amplitude. C) Additional examples of Venus::BMAL1 (top)/ PER2::Luc (bottom) parallel imaging in hip cartilage explants, where the paired PER2::Luc traces are below the corresponding Venus::BMAL1 trace. Data are in relation to Fig 3F, n = 3 mice. D) Real time confocal imaging of Venus::BMAL1 in hip cartilage tissue explant, in relation to Fig 3F. Note, associated close-up images highlight the plots of average intensity of the whole field (white box below the yellow BF image), as well as the background ROI outside the cartilage field (bottom left white box measured as control). E) Daily dynamics of Venus::BMAL1 in cultured chondrocytes following dexamethasone treatment. Fluorescence intensity of individual nuclei was calculated by nucleus-tracking and quantification. Individual autocorrelation traces for tracked Venus::BMAL1 chondrocytes are shown. Note the heterogeneity between individual cells. Out of the 236 cells, 105 showed a “circadian” (15-30h) period, 21 showed ultradian periodicities (0-15h) and 110 showed periodicities greater than 30 hours, and are thus described as “arrhythmic” because the total recording length was insufficient to accurately fit longer period rhythms. F) Frequency distribution histogram of Venus::BMAL1 periodicity exhibited by cells tracked in E). G) FLIP analysis to assess the nucleus/cytoplasm mobility of Venus::BMAL1 in unsynchronized MEFs and chondrocytes. The nuclear Venus::BMAL1 signal was continuously measured during bleaching in the cytoplasm. Nuclear signals from neighbouring unbleached cells were used as a control to normalise potential loss of signals over time. Data are shown as mean ±SEM, n = 3. H) Example images in relation to G, showing before (pre-bleach) and after (post-bleach) bleach of the cytoplasm (larger white outlines), where a portion of the nucleus of the same cell is monitored for loss of fluorescence (smaller white circles) and compared to unbleached cells (blue circles). Scale bar = 20 μm.
(TIF)

S5 Fig Venus::BMAL1 protein behaviour. A) Venus::BMAL1 half-life in mouse embryonic fibroblasts (MEFs). Cells were treated with CHX 5 μg/mL to stop *de novo* protein synthesis. Left: Individual curves showing fluorescence decay after CHX treatment in MEFs. Right: Box and whisker plot showing median and interquartile range for half-life of Venus::BMAL1 in MEFs (n = 25 cells). B) *Bmal1* mRNA stability in primary MEFs. Time (min) is following actinomycin D treatment (5 μg/mL). Related to Fig 4C. C) Raw data of Venus::BMAL1 recording in (left) SCN slices treated with CHX or vehicle and (right) chondrocytes. Note there is no acquisition bleaching in vehicle treated slices.
(TIF)

S6 Fig FCS correlations-fit deviation curves. Representative correlations- fit deviation curves for FCS measurements in MEFs and chondrocytes. Data from all cells fitted well to a one-component diffusion model with a triplet state.
(TIF)

S1 Movie Multi-spectral (lambda) imaging of primary chondrocytes. Spectral linear unmixing revealed nuclear localization of Venus::BMAL1, with most of the cytoplasmic fluorescence attributed to auto-fluorescence. Yellow: Venus::BMAL1; Red: CellMask™ Deep Red Plasma membrane stain; blue: auto-fluorescence.
(MOV)

S2 Movie Multi-spectral (lambda) imaging of primary MEFs. Spectral linear unmixing revealed nuclear localization of Venus::BMAL1, with most of the cytoplasmic fluorescence attributed to auto-fluorescence. Yellow: Venus::BMAL1; Red: CellMask™ Deep Red Plasma membrane stain; blue: auto-fluorescence.
(AVI)

S3 Movie Real-time live imaging of Venus::BMAL1 SCN organotypic slice. (MOV)

S4 Movie Real-time live imaging of Venus::BMAL1 in dispersed primary chondrocytes. (MOV)

S5 Movie Real-time imaging of Venus::BMAL1 in primary chondrocytes going through cell division. (MP4)
