# Supplementary figures and images for: Quantitative live imaging of Venus::BMAL1 in a mouse model reveals complex dynamics of the master circadian clock regulator
Source: PLoS Genet. 2020 Apr 30;16(4):e1008729. doi: 10.1371/journal.pgen.1008729 (PMC7217492; doi:10.1371/journal.pgen.1008729)

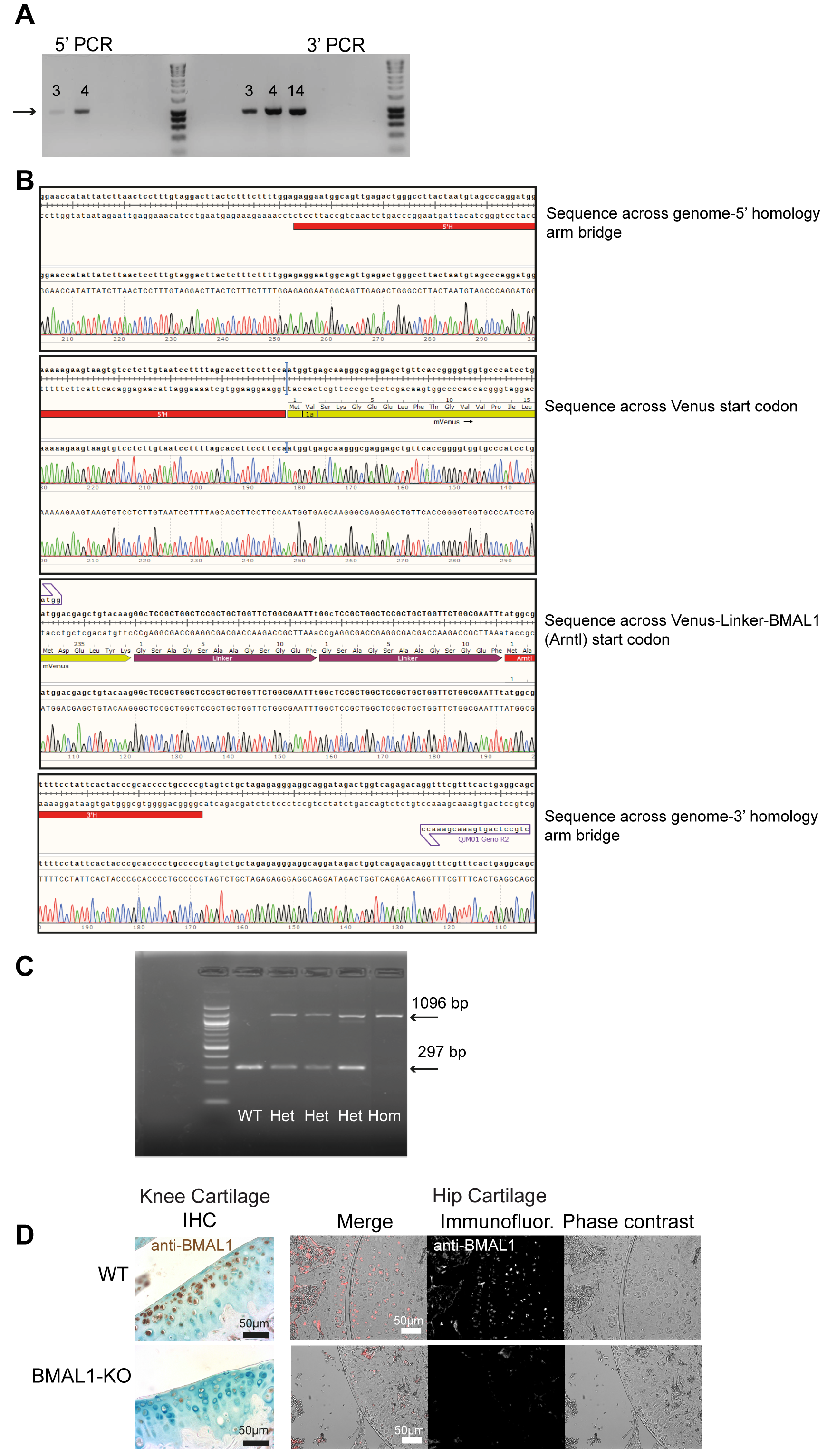

Supplement: S1 Fig — A) PCR analysis of 5’ (1050bp) and 3’ (970bp) genome-transgene integration. The number above each band denotes mouse ID. B) DNA sequencing to confirm the “bridge” junctions of the Venus-Bmal1 allele. All junctions were correct as predicted. C) PCR genotyping of breeding colonies indicating band patterns for wild type (WT), heterozygotes (Het) and homozygotes (Hom). WT allele - 297bp; Knock-in allele - 1096bp. D) Validation of BMAL1 antiserum in knee and hip femoral head cartilage from WT and Col2a1-Cre/Bmal1-/- KO mice. (Left panel) Immunohistochemistry: anti-BMAL1 shown in brown. (Right panels) Immunofluorescence: anti-BMAL1 shown in red in merged image and white in single channel image. Related to Fig 1C. (TIF) [file pgen.1008729.s001.tif]

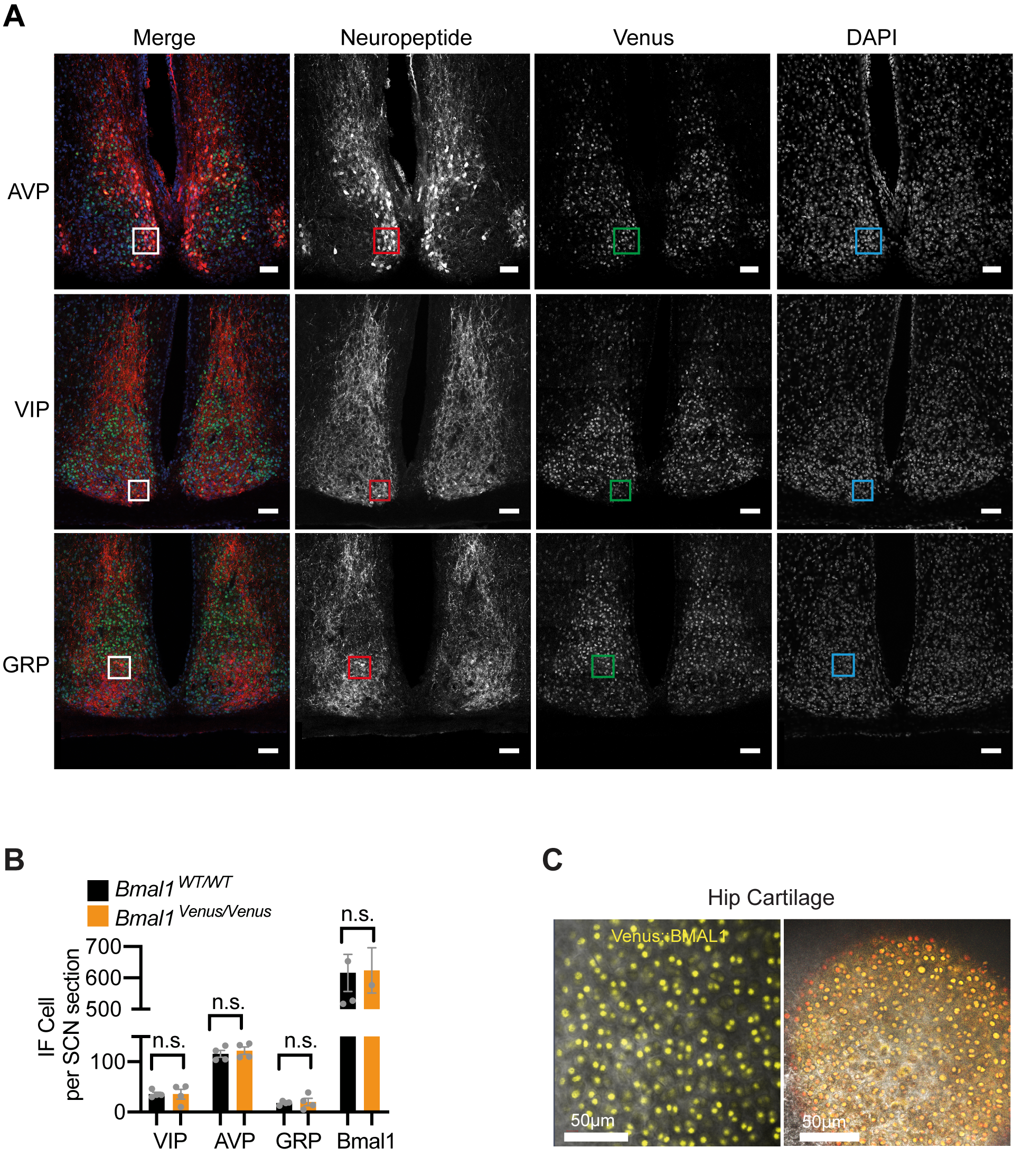

Supplement: S2 Fig — A) Low magnification of Venus::BMAL1 (green) co-localisation with neuropeptide-ir (red) in the SCN, co-stained with DAPI (blue). Scale bar = 100μm. Note that this is a composite image from 5x5 tile scan resulting in minor image-join artefacts (in relation to Fig 1E). B) SCN cell-type composition was determined by cell counts of different neuropeptide-expressing cells (n = 4-5 mice). Note there was no difference in the cellular composition between Venus and non-Venus mice. C) Left: representative confocal micrograph showing Venus::BMAL1 fluorescence in hip cartilage tissue explant. Right: a merged image of Venus and DRAQ5 shows nuclear localisation of Venus::BMAL1 in hip chondrocytes. (TIF) [file pgen.1008729.s002.tif]

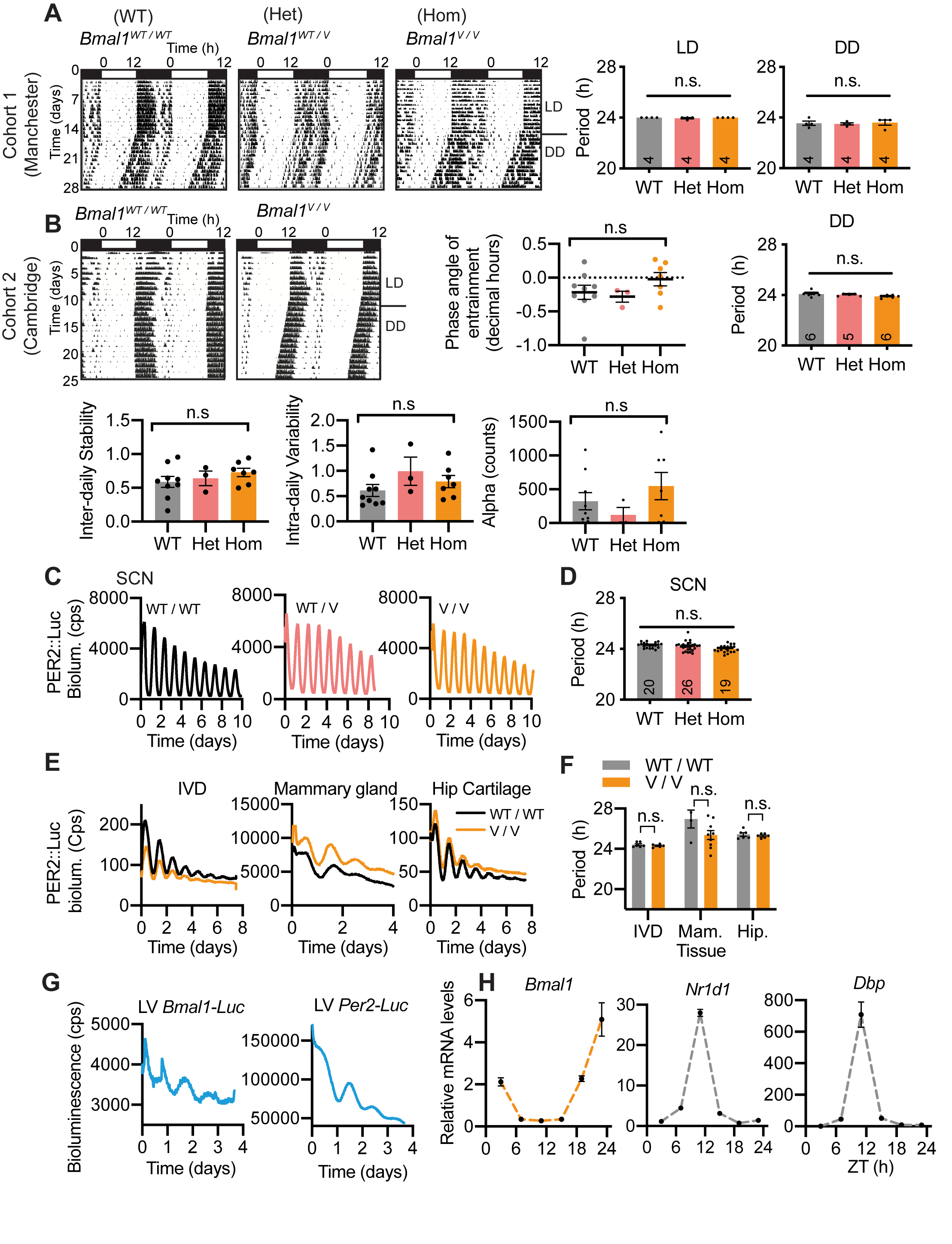

Supplement: S3 Fig — A, B) Representative double-plotted actograms showing normal wheel-running behaviour of wild type (WT/WT or WT), heterozygous (WT/V or Het) and homozygous (V/V or Hom) mice. Circadian periods for wheel-running during 12:12 light-dark (LD) cycling conditions and subsequent continuous darkness (DD) (n ≥4). There were no significant differences in phase of entrainment to the L/D cycle, the robustness of circadian behaviour (intra-daily variability and inter-daily stability non-parametric analyses), nor the duration of the main activity bout (alpha) (n >4; mean ±SEM). C) Representative PER2::Luc molecular rhythms in SCN organotypic slices. D) Mean ±SEM circadian periods for the SCN (n >18 for each group). E) Representative PER2::Luc rhythms in peripheral tissue slices (IVD, mammary gland and hip cartilage) of WT (black) and Venus::BMAL1 (orange) mice. F) Mean ±SEM circadian periods for peripheral tissues. G) Fibroblasts from Venus::BMAL1 mouse demonstrate normal circadian pacemaking as shown by antiphase oscillations of RORE- and E-Box-containing clock reporters. Bmal1-Luc or Per2-Luc reporters were transduced into primary Venus::BMAL1 MEFs by lentivirus. Cells were synchronised by dexamethasone before bioluminescence recording. H) Robust rhythmicity of endogenous clock genes in liver of Venus::BMAL1 mouse determined by qPCR. (TIF) [file pgen.1008729.s003.tif]

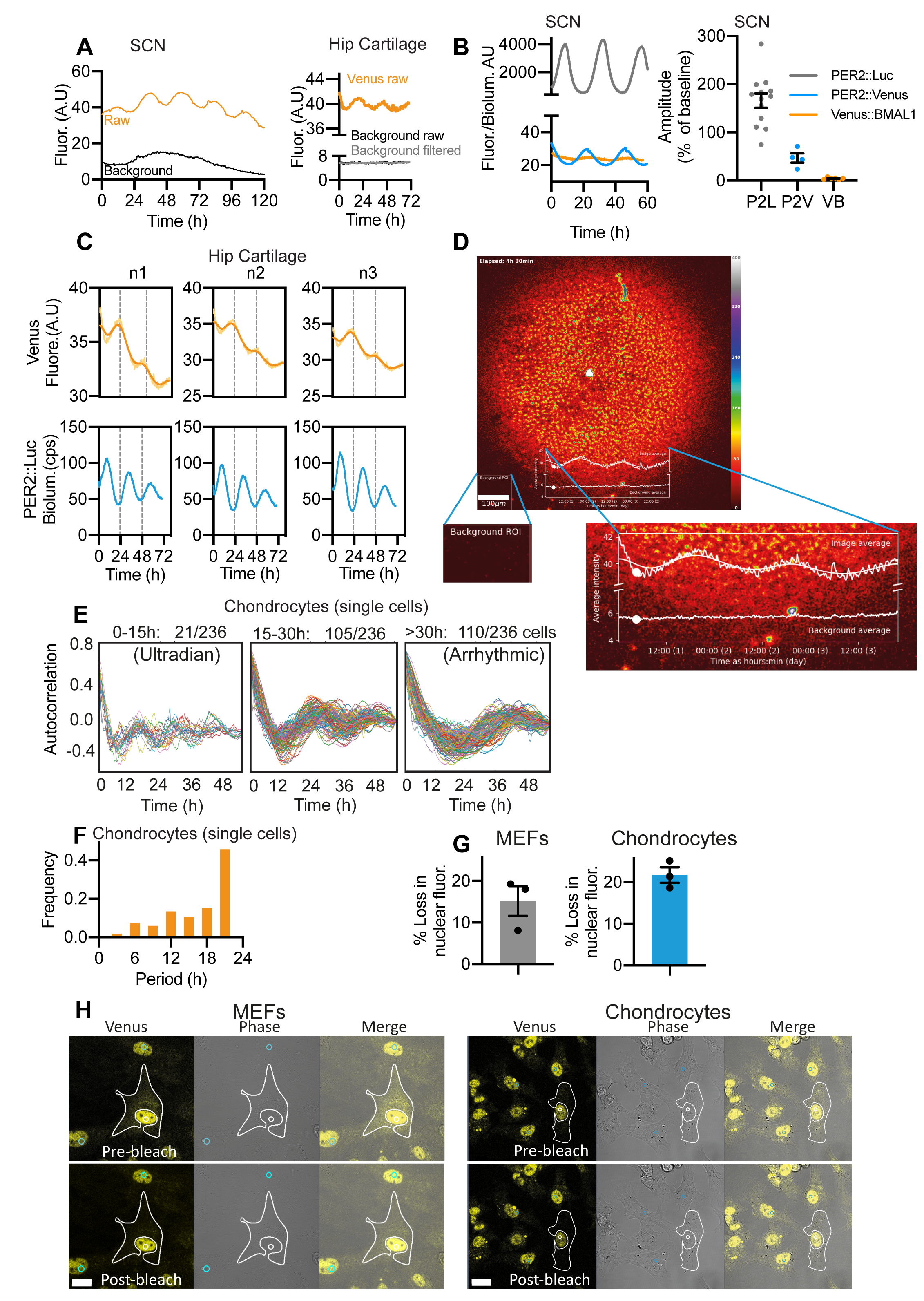

Supplement: S4 Fig — A) Average fluorescence recordings related to Fig 3A (left) and Fig 3F (right). Raw fluorescence intensities shown in orange and background fluorescence shown in black/grey. Background intensities are subtracted from raw intensities and shown as background subtracted, in the main figure. B) (Left) Representative raw traces from SCN timelapse recordings: PER2::Luc (grey), PER2::Venus (blue) and Venus::BMAL1 (orange) plotted on the same graph to compare their amplitudes of circadian oscillation. (Right) Group data (mean ± SEM) showing percentage amplitude (relative to baseline) for each reporter. Note that PER2::Venus and Venus::BMAL1 use the same fluorescence reporter so are most comparable in terms of assessing amplitude. C) Additional examples of Venus::BMAL1 (top)/ PER2::Luc (bottom) parallel imaging in hip cartilage explants, where the paired PER2::Luc traces are below the corresponding Venus::BMAL1 trace. Data are in relation to Fig 3F, n = 3 mice. D) Real time confocal imaging of Venus::BMAL1 in hip cartilage tissue explant, in relation to Fig 3F. Note, associated close-up images highlight the plots of average intensity of the whole field (white box below the yellow BF image), as well as the background ROI outside the cartilage field (bottom left white box measured as control). E) Daily dynamics of Venus::BMAL1 in cultured chondrocytes following dexamethasone treatment. Fluorescence intensity of individual nuclei was calculated by nucleus-tracking and quantification. Individual autocorrelation traces for tracked Venus::BMAL1 chondrocytes are shown. Note the heterogeneity between individual cells. Out of the 236 cells, 105 showed a “circadian” (15-30h) period, 21 showed ultradian periodicities (0-15h) and 110 showed periodicities greater than 30 hours, and are thus described as “arrhythmic” because the total recording length was insufficient to accurately fit longer period rhythms. F) Frequency distribution histogram of Venus::BMAL1 periodicity exhibited by cell [file pgen.1008729.s004.tif]

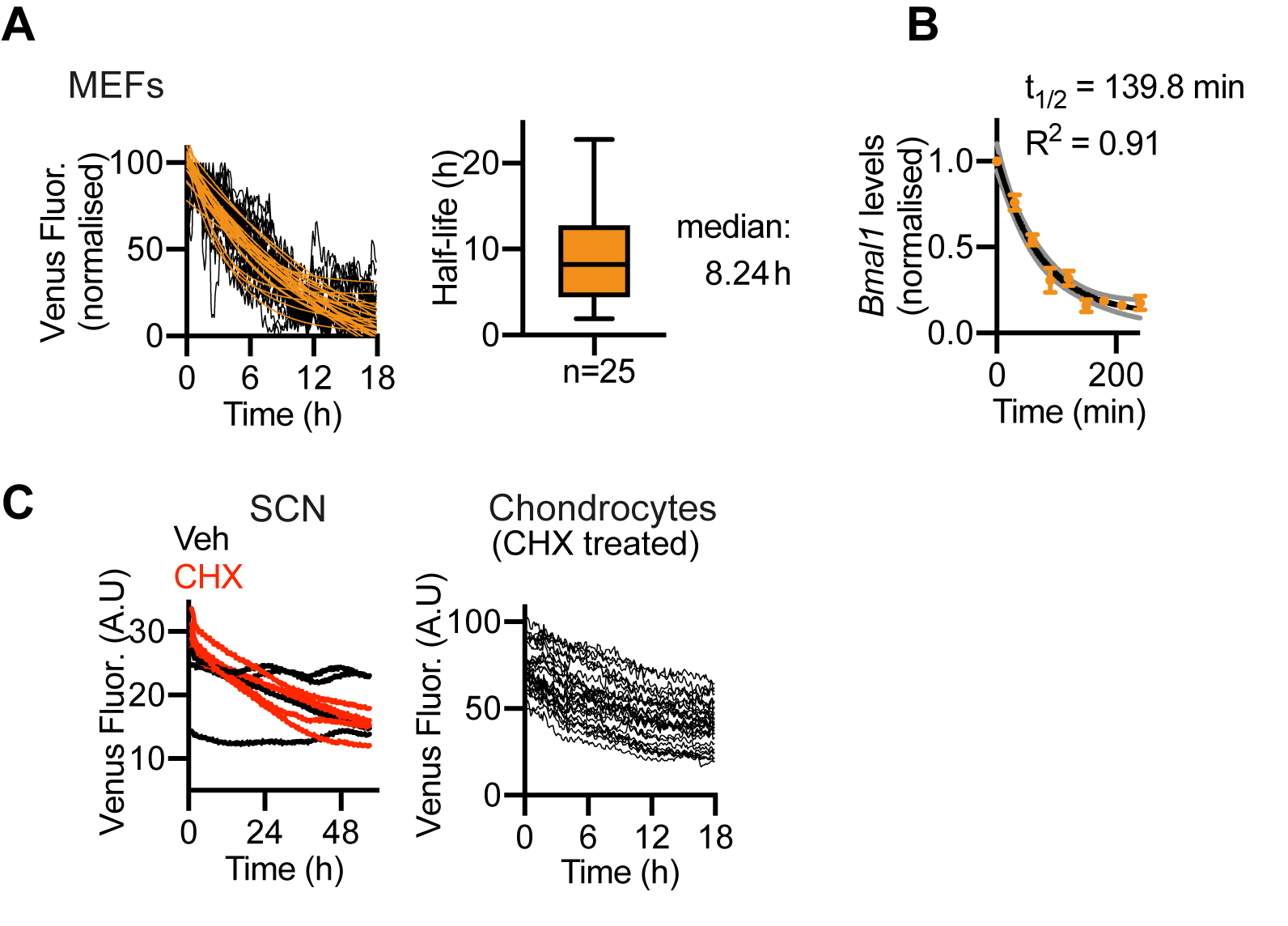

Supplement: S5 Fig — A) Venus::BMAL1 half-life in mouse embryonic fibroblasts (MEFs). Cells were treated with CHX 5 μg/mL to stop de novo protein synthesis. Left: Individual curves showing fluorescence decay after CHX treatment in MEFs. Right: Box and whisker plot showing median and interquartile range for half-life of Venus::BMAL1 in MEFs (n = 25 cells). B) Bmal1 mRNA stability in primary MEFs. Time (min) is following actinomycin D treatment (5 μg/mL). Related to Fig 4C. C) Raw data of Venus::BMAL1 recording in (left) SCN slices treated with CHX or vehicle and (right) chondrocytes. Note there is no acquisition bleaching in vehicle treated slices. (TIF) [file pgen.1008729.s005.tif]

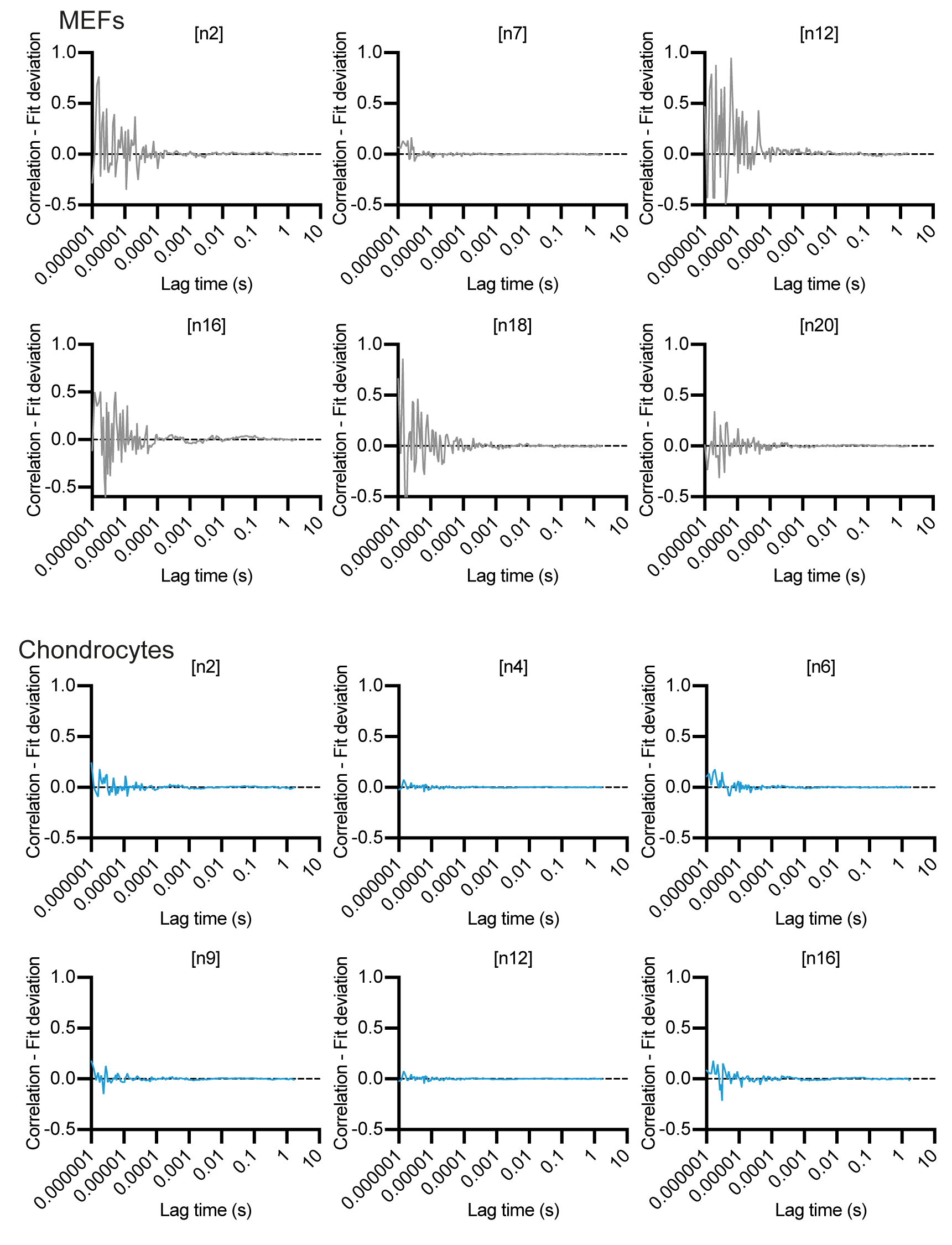

Supplement: S6 Fig — Representative correlations- fit deviation curves for FCS measurements in MEFs and chondrocytes. Data from all cells fitted well to a one-component diffusion model with a triplet state. (TIF) [file pgen.1008729.s006.tif]
